# Antifungal Activity and Stability of Fluconazole Emulsion Containing Ionic Liquids Explained by Intermolecular Interactions

**DOI:** 10.3390/pharmaceutics14040710

**Published:** 2022-03-26

**Authors:** Bruno L. Hennemann, Caroline R. Bender, Guilherme S. Moleta, Ânderson R. Carvalho, Luana C. G. Bazana, Alexandre M. Fuentefria, Clarissa P. Frizzo

**Affiliations:** 1 Department of Chemistry, Federal University of Santa Maria, Santa Maria 97105900, Brazil; bruno.l.h1@gmail.com (B.L.H.); guilhermescalcao@gmail.com (G.S.M.); 2 Department of Chemistry, Federal University of Pampa, São Gabriel 97307020, Brazil; carolinebender@unipampa.edu.br; 3 Laboratory of Applied Mycology, College of Pharmacy, Federal University of Rio Grande do Sul, Porto Alegre 90610000, Brazil; andersonrc87@gmail.com (Â.R.C.); luanacgb93@hotmail.com (L.C.G.B.); alexandre.fuentefria@ufrgs.br (A.M.F.)

**Keywords:** ionic liquid, ultrasound, emulsion, fluconazole, intermolecular interaction, antifungal activity

## Abstract

This research reports accelerated stability experiments, the evaluation of intermolecular interactions, and antifungal assays for fluconazole emulsions prepared using ultrasound (US) and magnetic stirring (MS) in the presence of ionic liquids derived from 1,*n*-(3-methylimidazolium-1-yl)alkane bromide ([C_n_MIM]Br; *n* = 12 or 16). The goals of the investigation are to quantify the stability, identify the forces that drive the formation and stability, and determine the antifungal activity of fluconazole-containing emulsions, and corroborate the data from our previous results that indicated that the emulsion based on [C_16_MIM]Br seemed to be more stable. In this study, accelerated stability experiments evidenced a considerable stability for the [C_16_MIM]Br emulsions at two temperatures (25 and 37 °C)—the instability index increased in the following order: US40% < US20% < MS. The ^1^H NMR data showed that the ILs interacts differently with medium-chain triglycerides (MCT). Two distinct interaction mechanisms were also observed for [C_12_MIM]Br and [C_16_MIM]Br with fluconazole, in which the latter formed more compact mixed aggregates than the former. The result was corroborated by diffusion data, which showed that ILs suffered a decrease in diffusion in the presence of fluconazole. The antifungal assay showed that emulsions containing ILs displayed superior activity compared with fluconazole alone. The emulsions also showed potent activity in inhibiting a resistant species (*C. glabrata*—CG34) to FLZ. All emulsions showed weak irritant potential in HET-CAM assay.

## 1. Introduction

**Figure 1:**
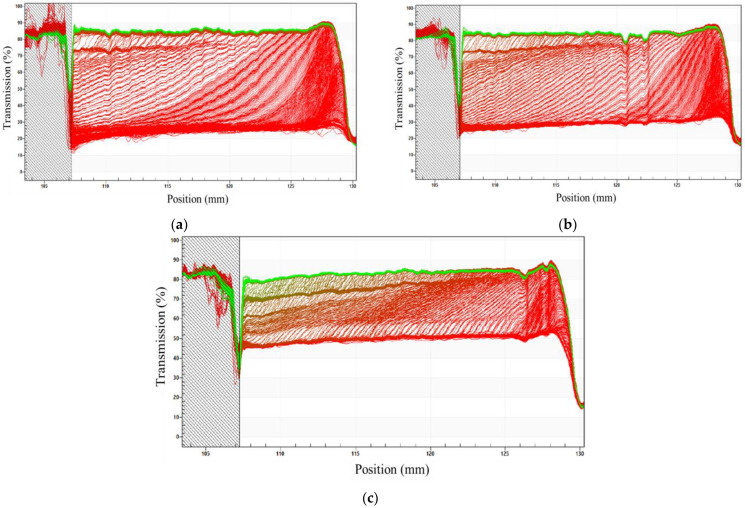
Transmission profiles as a function of the position in the cuvette for the [C_16_MIM]Br emulsions (3.1 mM, at 25 °C) prepared by (**a**) MS, (**b**) US 20%, and (**c**) US 40%.

Fluconazole (Figure 1a) is an antifungal agent against a broad spectrum of fungal species, including *Blastomyces*, *Candida*, *Coccidioides*, *Cryptococcus*, *Epidermophyton*, *Histoplasma*, *Microsporum*, and *Trichophyton* [1]. Nevertheless, this drug has solubility issues (i.e., soluble in organic solvents, not soluble in water), thus limiting the preparation of pharmaceutical formulations and their release in living organisms [1]. Fluconazole is currently commercialized as pills. However, this form hampers the bioavailability of the drug and, consequently, its action on the body specific target. To overcome these limitations, emulsions have been investigated as an alternative to stabilize fluconazole and enable its topical application [2].

To improve emulsion stability, some protocols have been proposed, including the utilization of imidazolium-based ILs as emulsifier agents rather than conventional surfactants [3,4]. Ionic liquids with a long lateral chain are charged surface-active species capable of forming micelles and, for this reason, are good candidates to replace uncharged surfactants since they can also confer additional advantages to the formulations [5]. This class of ILs was proven to be more effective in stabilizing emulsified systems. The advantages of ILs in an emulsion are their greatest attraction, with co-surfactants promoting better drug immobilization and greater emulsion stability. In addition to assisting the formation of conventional emulsions, ILs can also act in drug delivery systems as they can be used to increase the solubility of drugs with limited solubility and enhance their topical and transdermal delivery [4,6,7,8,9,10,11]. This notable performance is frequently attributed to the charged nature of amphiphilic ILs in relation to conventional not charged amphiphilic molecules. Furthermore, due to the possibility of changes being made to the cation, anion, and/or the alkyl chain of these ILs’ structures, their properties can be easily adjusted according to the limitation to be overcome [12,13,14,15].

In a previous study, we presented the encapsulation of fluconazole using the imidazolium-based ILs—[C_12_MIM]Br or [C_16_MIM]Br—through the formation of emulsions assisted by either US irradiation (at amplitudes of 20% and 40%) or magnetic stirring [16]. The results showed that the emulsion prepared using US at an amplitude of 40% resulted in increased encapsulation efficiency, lower viscosities, and better control of drug release. Additionally, emulsions based on [C_16_MIM]Br presented a smaller droplet size with a spherical shape and smaller polydispersity (span index), indicating increased preliminary stability.

Emulsion stability can be investigated through thermal, chemical, and photophysical properties. Among these, the most studied is photophysical stability, which can be assessed in two steps: preliminary and accelerated stability. The preliminary test consists of assessing the emulsions visual control, particle size measurement, zeta potential, and polydispersity index. Accelerated stability studies are based on techniques that stimulate emulsion instability (e.g., centrifugation) and are of fundamental importance to understand the reliability and reproducibility of the prepared emulsions [17,18,19,20].

As shown in our previous study, [C_16_MIM]Br emulsions need more energy in order to be formed compared to [C_12_MIM]Br. Moreover, the former yields emulsions that are more stable than those prepared using the latter. Therefore, advances in stability tests and a better understanding of the driving forces behind the formation of medium-chain triglycerides (MCTs) and fluconazole emulsions using [C_12_MIM]Br and [C_16_MIM]Br (Figure 1b) are highly desirable. The study presented herein aims to support our previous results on emulsion formation using imidazolium-based ILs, which indicate a greater stability of emulsions formed by [C_16_MIM]Br compared to those prepared using [C_12_MIM]Br. This work provides IL solution diffusion data in the presence of MCT or fluconazole to help explain which intermolecular interactions are involved in each case and their relationship with emulsion formation and stability.

To achieve this, the chemical shifts in the IL nuclei and diffusion coefficients were monitored by ^1^H NMR experiments, respectively, in the presence and absence of fluconazole or MCT. Accelerated physical stability was determined using a High-End Dispersion analyzer at 25 °C and 37 °C. Additionally, to verify the impact of an emulsion on the antifungal activity of fluconazole, considering the possible value of incorporating these drugs in topical formulations, irritability tests were performed. 

## 2. Experimental Section

### 2.1. Materials

1-bromohexadecane, 1-bromododecane, 1-methylimidazole, and fluconazole were purchased from Sigma-Aldrich (St. Louis, MO, USA). Acetone (HPLC) was purchased from Vetec (Duque de Caxias, RJ, Brazil) and was used without further purification. Medium-chain triglyceride (MCT) with an HLB of 5 was purchased from Ceres^®^ MCT-oil (Weßling, Germany).

### 2.2. Synthesis

The ILs were synthesized following the methodology previously described in the literature and adapted in our laboratory [21,22]; the updated synthesis methodology is reported in reference [16]. All ILs were characterized by electrospray ionization mass spectrometry (ESI-MS) and ^1^H and ^13^C nuclear magnetic resonance (NMR) spectroscopy using D_2_O as a solvent [16].

### 2.3. Preparation of Emulsions

Emulsion preparation via magnetic stirring (MS) and ultrasound (US) and characterization data (e.g., polydispersity and viscosity) are described in detail in reference [16].

### 2.4. NMR

NMR spectra were recorded on a Bruker AVANCE III 600 (^1^H at 600.13 MHz) spectrophotometer equipped with a BCU II unit to heat/cool the probe (temperature range: 193.15–333.15 K). Emulsion samples and separated components were placed in 5 mm tubes. For the NMR experiments, emulsion samples were prepared directly in D_2_O. Chemical shifts (δ) in ppm were related to tetramethylsilane (TMS) as an external reference. The temperature of the experiments was 25 °C, and the digital resolution was ±0.18 Hz/point. The number of scans was 16, and water suppression was not necessary.

### 2.5. DOSY-NMR

The experiments involving diffusion (D) were performed at 298 K on a Bruker Avance III (^1^H at 600.13 MHz) equipped with a BCU II unit to heat and cool the probe (temperature range: 193.15–333.15 K). We used 5 mm NMR tubes containing D_2_O solutions of [C_12_MIM]Br and [C_16_MIM]Br at concentrations of 169 mM and 62 mM, respectively, in the presence of a sealed capillary tube with TMS diluted in CDCl_3_ as an external reference (digital resolution of ±0.01 ppm). For the spectra in the presence of TCM, successive additions of oil were performed in the ILs-D_2_O solutions in the following volumes: 0.05, 0.1, and 0.15 mL. For the spectra of the ILs in the presence of fluconazole mixtures, 8 mg of fluconazole was added to the ILs-D_2_O solutions previously prepared. Chemical shifts are expressed in ppm.

The self-diffusion coefficient (D) was obtained via the pulsed gradient spin echo (PGSE) method. The pulse sequence STEBPGP1S was used for samples at 25 °C. Values of 1800 µs and 0.05 s were used for the p30 and d20 parameters, respectively. A gradient amplitude of 2–98% and 16 scans were used. These experiments were performed to evaluate the intermolecular interactions between the components of the emulsions. 

### 2.6. Determination of Emulsion Stability

Initially, 200 µL of the emulsion was added to polycarbonate tubes with the aid of a syringe. Subsequently, the tubes were placed in a high-end dispersion analyzer (LUMiSizer^®^) and centrifuged at 2000 rpm (around 530 to 533× *g*) for 10 h. The analyses were conducted at 25 °C and 37 °C, with the latter being selected to simulate the human body temperature. From the passage of a laser with multiple wavelengths (λ) through the samples, the transmittance was measured in the emulsions over 10 h. At the end of the experiment, the phase separation of the emulsions was performed. With this technique, emulsion stability as well as a better understanding of destabilization kinetics was possible. 

### 2.7. Biological Evaluation

#### 2.7.1. Strains and Growth Media

Two strains, CG34 (*Candida glabrata*) and CAMS1 (*Candida albicans*) were selected to obtain an inhibitory concentration of 50% (IC50). Both yeasts used in this study underwent identification by MALDI-TOF. The selected yeasts were grown in SDA (São José dos Pinhais, PR, Brazil) at 35 °C for 24 h before the inoculum preparation. The antimicrobial sensitivity test (AST) was performed using RPMI-1640 culture medium (Waltham, MA, USA), buffered with 0.165 M MOPS, supplemented with 0.03% (*w/v*) l-glutamine, and 2% (*w/v*) d-glucose. The medium was adjusted to pH 7.0.

#### 2.7.2. Antimicrobial Sensitivity Test (AST)

AST was performed by broth microdilution methodology, following the EUCAST E.DEF 7.3.2 protocol [23]. The strains were tested for four nanoemulsions described later containing 0.5 mg mL^−1^ of fluconazole (FLZ). The same four nanoemulsions without the FLZ antifungal incorporated were also tested as “background”. The commercial antifungal Fluconazole (Itapevi, SP, Brazil) was used as an AST control to compare the efficacy of nanoemulsions. ASTs were read 24 h after incubation at 35 °C using a plate reader (São José, SC, Brazil) at the absorbance of 620 nm (wavelength that does not suffer interference from the indicator); the optical density was used to obtain an inhibitory IC_50_ and IC_90_ using the GraphPad Prism 8 software (GraphPad software Inc., La Jolla, CA, USA). Data were normalized, and antifungal concentrations were log-transformed. The inhibition dose–response curves were obtained using the log (inhibitor) vs. normalized response—variable slope model.

### 2.8. Hen’s Egg Test on the Chorioallantoic Membrane (HET-CAM)

Fresh, white, fertile Lohmann eggs (Lohmann selected Leghorn, LSL) were used in the HET-CAM test. The eggs were kept optimized at incubation conditions (temperature between 38 and 39 °C and humidity between 55% and 60% for 10 days). On the 10th day, the egg shell around the airspace, was carefully removed with a rotary tool (Racine, WI, USA). Afterwards, 0.3 mL of each substance was added to each egg, respectively (negative control—0.9% saline solution; positive control—0.1 M NaOH solution, fluconazole and emulsions). The irritant effect was observed at times of 30 s, 2 min, and 5 min after the application of each substance. The result of the irritation score (IS) was given according to the equation below, on a scale from 0 to 4.9 denoting non-irritant (or practically no irritation) and 5.0 to 21 denoting irritant (moderate/severe or extreme irritation) (ICCVAM, 2010)

$$IS=\left(\left(\frac{\left(301-Hemorrhage\;Time\right)}{300}\right)\times 5\right)+\left(\left(\frac{\left(301-Lysis\;Time\right)}{300}\right)\times 7\right)\;+\left(\left(\frac{\left(301-Coagulation\;Time\right)}{300}\right)\times 9\right)$$

### 2.9. Statistical Analysis

Differences between inhibitory concentrations (IC_50_) were performed through the t-test, and the threshold of *p* < 0.05 was considered significant. The one-way ANOVA was used to evaluate the differences between groups in the HET-CAM assay followed by the Tukey test, with a threshold of *p* < 0.05 for significant results.

## 3. Results and Discussion

The stability of the emulsions (at three concentrations) was investigated using accelerated experiments at two temperatures (25 °C and 37 °C), and a series of ^1^H NMR experiments were subsequently performed. 

### 3.1. Emulsion Stability Tests

The physical stability of the IL emulsions containing fluconazole, prepared using mechanical stirring (MS) and ultrasound (US) at power 20% and US 40%, was assessed at 25 °C and 37 °C (body temperature) using the LUMiSizer^®^ analyzer. This multi-sample analytical instrument employs centrifugal sedimentation to accelerate the occurrence of instability phenomena such as sedimentation, flocculation, or creaminess [24,25]. The equipment allows for the measurement of the intensity of light transmission as a function of time and position over the entire sample, known as the “cream rate”. This rate is related to the physical stability of the emulsion; the lower the cream rate, the greater the stability [26]. Figure 1 shows the transmission profiles of emulsions at 25 °C with 3.1 mM of [C_16_MIM]Br prepared by MS, US 20%, and US 40%. 

**Figure 2:**
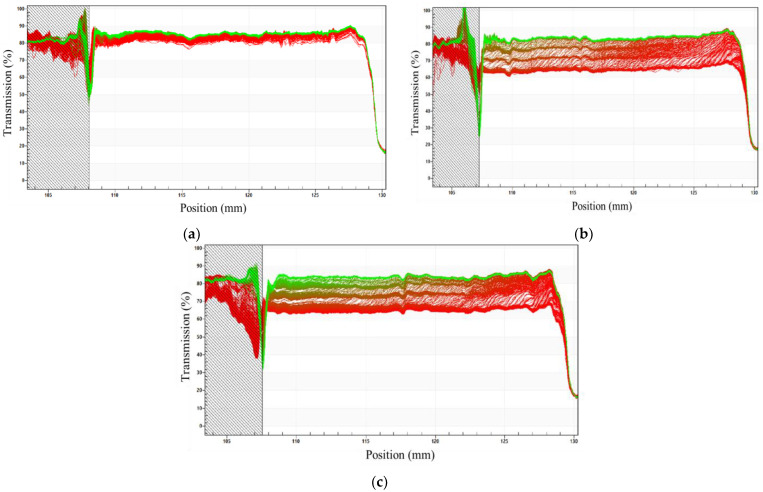
Transmission profiles as a function of the position in the cuvette for the [C_12_MIM]Br emulsions (3.6 mM, at 25 °C) prepared by (**a**) MS, (**b**) US 20%, and (**c**) US 40%.

The results showed that the sample prepared using MS (Figure 1a) had the lowest stability among the emulsions. The difference between the first (red) and last (green) transmission profiles was large, indicating the instability of this system. Furthermore, by analyzing the transmission profiles of the curves, it was found that at values close to 125 mm (lower part of the cuvette), the transmission is greater, which indicates that the phenomenon of destabilization occurring is creaming. This occurred for all emulsions based on [C_16_MIM]Br, regardless of the preparation mode. Creaming is characterized by gravitational separation, in which the drops move upward because they have a lower density than the continuous phase [27]. The trend in Figure 1b was also observed for emulsions with lower concentrations of IL ([C_16_MIM]Br at 25 °C), which indicates that the stability of these systems increases when the emulsions are prepared using higher energy (US 40%). A trend was also observed for emulsions at 37 °C. Transmission profiles for all emulsions at 25 °C and 37 °C are presented in Figures S1–S30 in the Supplementary Materials. The same experiments were performed on 3.6 mM [C_12_MIM]Br emulsions prepared by MS, US 20%, and US 40% (Figure 2).

**Figure 3:**
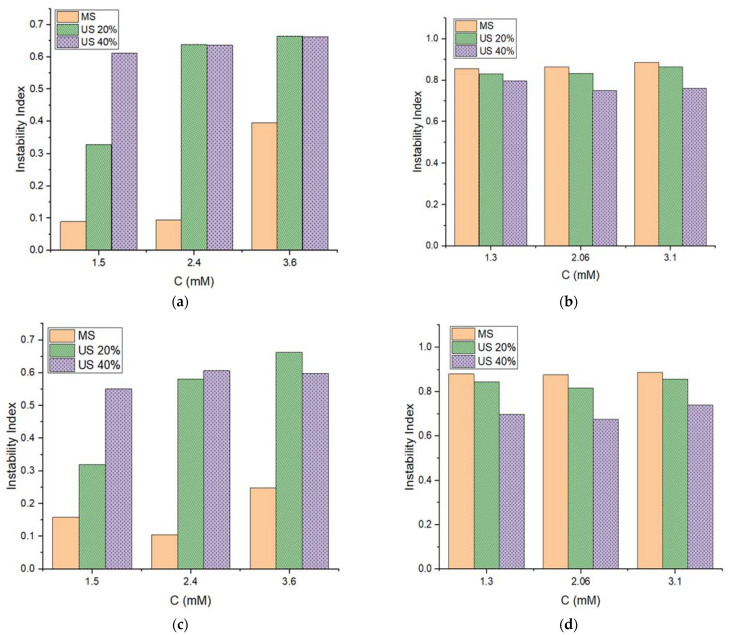
Instability indices for emulsions based on (**a**,**c**) [C_12_MIM]Br and (**b**,**d**) [C_16_MIM]Br at different IL concentrations (C) at 25 °C (**top**) and 37 °C (**bottom**).

As shown in Figure 2, all emulsions have transmission profiles that do not depend on temperature (25 °C and 37 °C) or the concentration of [C_12_MIM]Br. The trend displays a small variation in the transmittance of emulsions prepared using MS compared to those prepared at 20% and 40% US. This indicates that the high-energy method renders less stable [C_12_MIM]Br emulsions. Similar to what was observed for the emulsion based on [C_16_MIM]Br, a greater transmission was detected at values close to 127 mm (lower part of the cuvette), indicating the occurrence of creaming. Comparing the transmission profiles of the emulsions based on [C_12_MIM]Br and [C_16_MIM]Br, it is evident that the difference between the first (red) and the last (green) profiles for [C_12_MIM]Br is smaller than the difference for [C_16_MIM]Br. All transmission profiles of emulsions are shown in Figures S1–S30 in the Supplementary Materials. In addition to the aforementioned transmission profiles, LUMiSizer also provides instability indices for the systems (see Tables S1 and S2 in the Supplementary Materials). The indices for the studied emulsions are shown in Figure 3.

**Figure 4:**
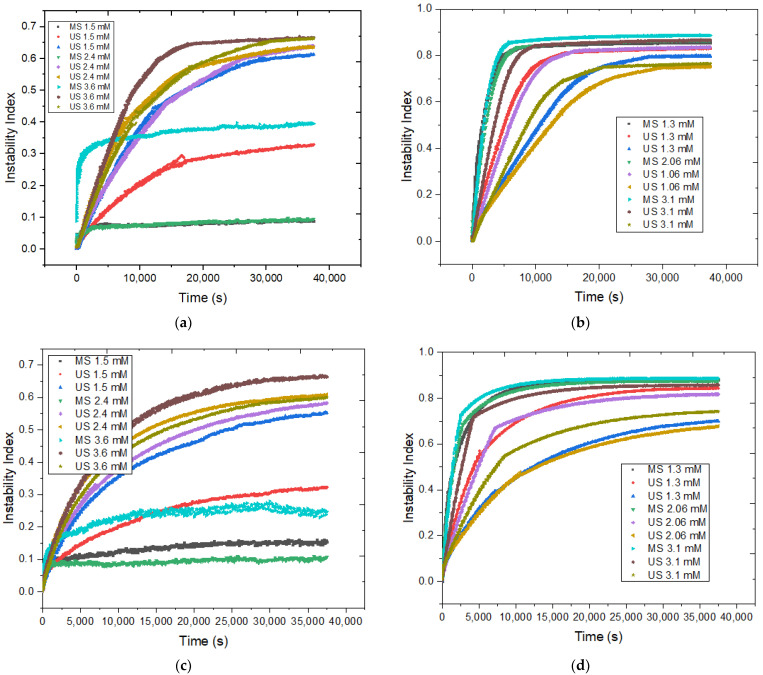
Instability index graphs for emulsions based on (**a**,**c**) [C_12_MIM]Br and (**b**,**d**) [C_16_MIM]Br at 25 °C (**top**) and 37 °C (**bottom**).

For the emulsions prepared with [C_12_MIM]Br (Figure 3a,c), a higher preparation energy led to an increase in the instability index. This observation agrees with our previous results on the preparation and characterization of these emulsions, where we found that increased preparation energy caused emulsion destabilization. Considering the concentration of [C_12_MIM]Br, it was observed that emulsions with 1.5 mM of IL prepared using MS and 20% US had a lower instability index compared to their analogues prepared with 2.4 and 3.6 mM of IL. Therefore, a smaller amount of IL was found to efficiently stabilize the emulsions (Figure 3b,d). For emulsions based on [C_16_MIM]Br, the instability index increased in the following order: US40% < US20% < MS. For these emulsions, the dependence on stability with the concentration of IL was not observed. Furthermore, no increase in the instability of [C_12_MIM]Br and [C_16_MIM]Br emulsions was detected when the temperature increased from 25 °C to 37 °C, indicating that these emulsions are favorable for potential applications at body temperature. Graphs of instability indices as a function of centrifugation time were also generated (Figure 4) for the evaluation of the destabilization kinetics, in which the sedimentation velocity is given by the slope of the curves [21]. The experiments were carried out at 25 °C and 37 °C.

**Figure 5:**
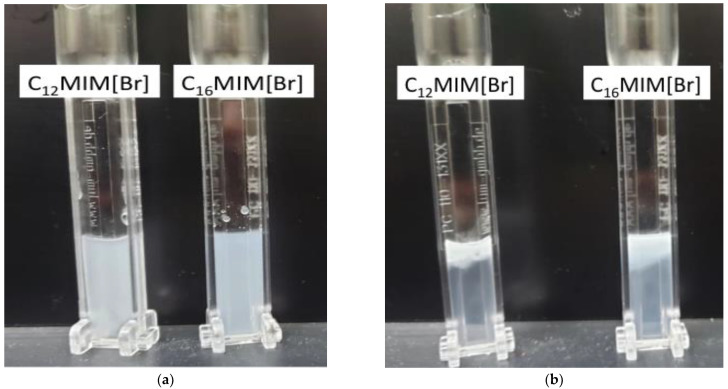
Visualization of [C_12_MIM]Br and [C_16_MIM]Br emulsions prepared using US 20% (**a**) before and (**b**) after centrifugation.

These results show that [C_12_MIM]Br emulsions at 1.5 mM, prepared using MS and 20% US at 25 °C and 37 °C, were destabilized more slowly than other emulsions based on this IL. The [C_16_MIM]Br emulsions prepared using 40% US (light gray, gray, and green lines in Figure 4b,d) destabilized more slowly than the other emulsions prepared using this IL. It is also evident that the slopes of the curves of emulsions based on [C_16_MIM]Br prepared using US are smaller than those for the other emulsions. Therefore, high concentration colloidal particles prepared using high US amplitudes can be more stable and difficult to break down because they contain a larger amount of [C_16_MIM]Br per droplet and many more hydrophobic interactions (London dispersion) are involved. It is reasonable that the steric barrier of the [C_16_MIM]Br chains is overcome at high US amplitudes (40%) in order to achieve the maximum critical micellar concentration (CMC) of [C_16_MIM]Br in the colloidal particles. A visual analysis of the emulsions after data acquisition in the LUMiSizer experiments confirmed that the destabilization of the emulsions occurred simultaneously with creaming. Figure 5 shows images of [C_12_MIM]Br and [C_16_MIM]Br emulsions prepared using 20% US before and after the LUMiSizer experiments, clearly showing that centrifugation accelerates the destabilization process.

### 3.2. Intermolecular Interactions between the Emulsion Components

**Figure 6:**
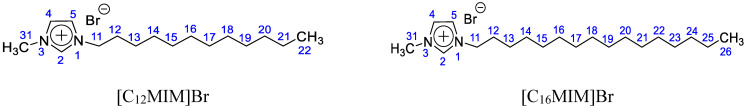
Schematic structures of imidazolium-based ILs used in this study.

The occurrence of intermolecular interactions between the emulsion components was determined using titration monitored by ^1^H NMR and ^1^H DOSY experiments (see Figures S31 and S32 in the Supplementary Materials of pure ILs ^1^H NMR spectra). The determination of diffusion coefficients (D) from the ^1^H DOSY of molecules in a solution is a fundamental tool for understanding the emulsion structure and dynamics [28]. ^1^H DOSY experiments for mixtures of ILs with MCT ILs with fluconazole were performed. For the experiments involving MCT, D values were determined for ILs in the absence and in the presence of MCT at varying quantities (0.05 mL, 0.1 mL, 0.15 mL, and 0.20 mL). The signals H11, H31, and H22 for [C_12_MIM]Br and H11, H31, and H26 for [C_16_MIM]Br were monitored as they do not overlap with other signals. The diffusion curves present the expected sigmoidal decay wherein the last point decreases between 5% and 15% in relation to the first point. Table 1 presents the diffusion coefficient (D) and average values obtained for [C_12_MIM]Br and [C_16_MIM]Br in the absence and presence of MCT. The ^1^H DOSY curves generated from the integration of the H11, H22, H26, and H31 signals from pure ILs and in the presence of MCT are presented in Figure S33 in the Supplementary Materials. The structure of ILs with the numbered nuclei are depicted in Figure 6.

It is evident that D values decreased as the MCT concentration increased for the hydrogen atoms of both [C_12_MIM]Br and [C_16_MIM]Br ILs. The average value follows the same trend, which is expected since a homogeneous system tends to diffuse equally. MCT is more viscous than the IL aqueous solutions; therefore, it is expected that the D values of ILs decrease when MCT is added to the systems. The single homogeneous phase of the systems that is characterized by the proportional decrease in D with the increasing concentration of MCT indicates that the components interact with each other. Otherwise, two phases would be formed because MCT is not soluble in water, and the chemical environment of the ILs would not be altered.

A similar methodology was used to determine the D values for both [C_12_MIM]Br and [C_16_MIM]Br in the presence and absence of fluconazole. H11, H31, H4, and H5 were monitored for all the molecules, while H22 and H26 were monitored for [C_12_MIM]Br and [C_16_MIM]Br. The obtained D values are shown in Table 2. The DOSY curves for H22 ([C_12_MIM]Br) and H26 ([C_16_MIM]Br), both in the absence and presence of fluconazole are presented in Figure S34 in the Supplementary Materials.

The results showed that when fluconazole was added to the systems, the D values decreased, thus indicating a modification in the movement of the molecule; this was probably due to the establishment of intermolecular interactions between the ILs and fluconazole molecules. An increase in the D of H4 and H5 of [C_12_MIM]Br was observed in the presence of fluconazole. This result suggests that these hydrogen atoms diffused more freely when fluconazole was added to the system. Presumably, the rearrangement of the IL molecules promoted by the encapsulation of fluconazole contributed to disruptions in the IL self-assembled micelles, which are stabilized by IL–IL intermolecular interactions in the aqueous environment. In contrast, it was found that in the case of [C_16_MIM]Br, the D values for H26 and H31 increased in the presence of fluconazole, while H11, H4, and H5 decreased. These results show that despite the similarity in the IL structures, the encapsulation of fluconazole by the IL with a longer alkyl side chain, [C_16_MIM]Br, resulted in a different behavior compared to the system stabilized by [C_12_MIM]Br.

**Figure 7:**
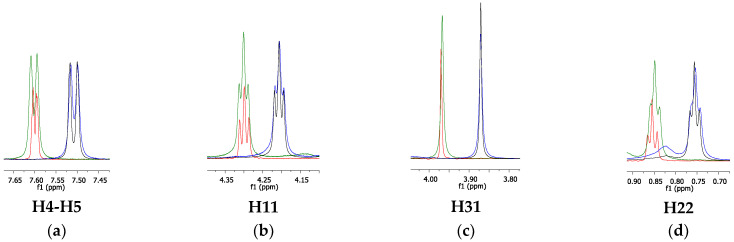
Overlap of the ^1^H signal of spectra for the following: (**a**) pristine [C_12_MIM]Br (red line), (**b**) [C_12_MIM]Br + 0.05 mL MCT (green line), (**c**) [C_12_MIM]Br + 0.10 mL MCT (blue line), and (**d**) [C_12_MIM]Br + 0.15 mL MCT (black line), respectively.

To obtain further information on the interactions between the emulsion components, ^1^H NMR spectra were also acquired for pristine ILs and in the presence of increased concentrations of MCT [29,30]. Spectra in the presence of fluconazole were not obtained because of the low solubility of this drug in water [31]. The ^1^H NMR spectra of [C_12_MIM]Br (169 mM) and [C_16_MIM]Br (62 mM) in D_2_O were recorded at much higher concentrations than the CMC of these ILs to ensure signal detection. The CMC values for [C_12_MIM]Br and [C_16_MIM]Br are 8.5 mM and 0.6 mM, respectively [32]. The spectra of the pure ILs are presented in the ESI. For the triplicate titration experiments, MCT was added to the IL solutions. Figure 7 displays the overlap of the ^1^H spectra of [C_12_MIM]Br in the absence and presence of MCT (0.05 mL, 0.10 mL, and 0.15 mL).

From Figure 7, it can be observed that the signal multiplicities were slightly attenuated in the presence of MCT. This multiplicity variation in the presence of MCT was previously observed by Li et al. [33] for emulsions prepared using [C_12_MIM]Br and *p*-xylene as the oil phase. The authors attributed this change in multiplicity to cation–π interactions between imidazolium and the aromatic ring of *p*-xylene. In this investigation, the change in the signal multiplicity suggests the occurrence of interactions between the imidazolium cations (of electron acceptors) of the ILs and the oxygen atoms of the ester groups of MCT (electron donors).

**Figure 8:**
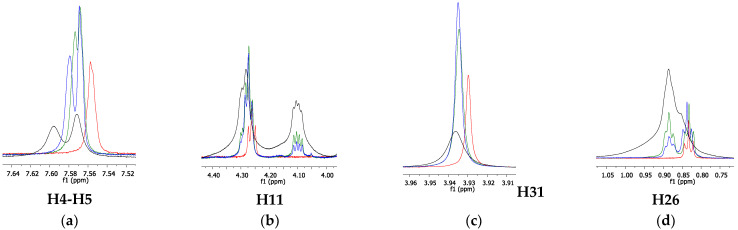
Overlap of the ^1^H signals of interest for the following systems: (**a**) pristine [C_16_MIM]Br (red line), (**b**) [C_16_MIM]Br + 0.05 mL MCT (green line), (**c**) [C_16_MIM]Br + 0.10 mL MCT (blue line), and (**d**) [C_16_MIM]Br + 0.15 mL MCT (black line), respectively.

As expected, the chemical shifts of the [C_12_MIM]Br signals were changed by the presence of MCT (Figure 7). The variation was more pronounced at greater concentrations, in which all the IL signals were shielded in the presence of 0.10 and 0.15 mL of MCT. This behavior can be attributed to the decrease in the polarity of the microenvironment MCT [34]. This result suggests that MCT molecules are inserted into the IL hydrophobic micelles, and they interact with the alkyl side chain of [C_12_MIM]Br. More specifically, H4-H5 and H31 likely interact via CH•••π, in which the IL hydrogen atoms are in the shielding cone of the MCT carbonyl and/or neighboring aromatic imidazolium rings from IL cationic heads. In addition, a change in the chemical environment of the hydrogen atoms at the end of the alkyl side chain (H22) was detected, thereby suggesting the establishment of van der Waals interactions. For the lower MCT concentration (0.05 mL), the IL signals almost did not change since the concentration of MCT was too low to cause a significant change in the average resonance signals for all IL monomers. These results are in accordance with the ^1^H DOSY experiments, in which only greater concentrations (e.g., 0.15 mM of MCT) caused a significant decrease in diffusion values. The same titration experiments were performed with [C_16_MIM]Br; Figure 8 shows the overlap of the ^1^H NMR signals for this IL in the absence and in the presence of MCT.

Figure 8 shows that [C_16_MIM]Br behaves differently than [C_12_MIM]Br in the presence of MCT. In this case, IL hydrogen atoms from the cationic head (H4-H5 and H31) deshield in relation to the pristine IL in the solution from the first MCT addition. The deshielding effect of the signals in the IL cationic heads can be related to the establishment of hydrogen bonds with MCT; the hydrogen atoms from the IL cationic heads and the MCT oxygen atoms and/or bromide anions act as hydrogen bond donors and acceptors, respectively. A split of the singlet attributed to H4-H5 for the pristine IL was observed in the presence of MCT. This result is similar to that of a previous study by Li et al. [33], which attributed this phenomenon to cation–π or π–π interactions between the IL imidazolium rings.

For both H11 and H22, the signals split in the presence of MCT from the IL alkyl side chain (Figure 8). This signal splitting can be attributed to the increase in the free energy of the system in the presence of MCT. As such, a reorganization of the IL micelles is required, thus allowing for the formation of MCT/IL mixed aggregates; this decreases the energy of the system. Nevertheless, this new assembly probably contains fewer IL monomers because of the presence of encapsulated MCT molecules; this results in free IL micelles in the solution. This could explain the similarity in the chemical shift for H31 in the emulsion and the pristine IL; the latter shows a lower δ. The deshielded signals of H11 and H26 (greater δ) nuclei are related to the formation of less compact MCT/IL aggregates than the pure IL micelles. These changes were pronounced for all IL hydrogen signals in the spectra, supporting the interaction between [C_16_MIM]Br and MCT. 

**Figure 9:**
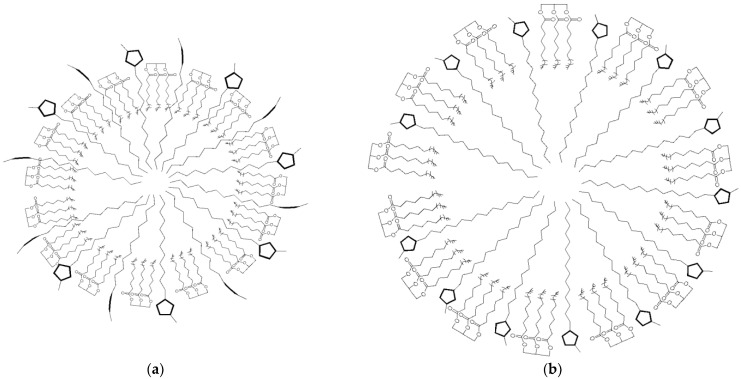
Schematic representation of mixed aggregates assembled by (**a**) [C_12_MIM]Br and (**b**) [C_16_MIM]Br with MCT.

Differences in the chemical shifts suggest the establishment of hydrogen bonds between the hydrophilic portions of ILs and MCT (oxygens from esters), as well as van der Waals interactions involving the IL alkyl side chains. This result indicates the more complex nature of the mixed aggregates of [C_16_MIM]Br in the presence of MCT. Visually, the addition of MCT to [C_16_MIM]Br forms a turbid and homogeneous mixture (see Figure S35 in the Supplementary Materials), characterizing the efficient incorporation of MCT into the aggregates. Figure 9 shows a schematic representation of the possible spherical aggregates assembled by ILs and MCT.

**Figure 10:**
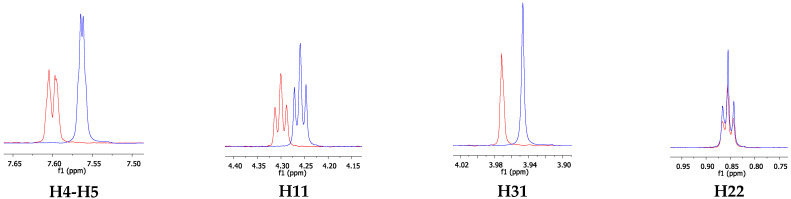
^1^H NMR spectra overlapping the expanded regions of interest for [C_12_MIM]Br in the absence (red) and in the presence of fluconazole (blue).

^1^H NMR experiments were also performed on ILs in the presence of fluconazole. Figure 10 shows the overlap of the ^1^H NMR spectra of [C_12_MIM]Br and [C_16_MIM]Br in the absence and in the presence of fluconazole.

**Figure 11:**
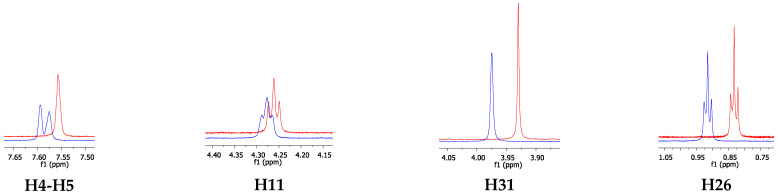
^1^H NMR spectra overlapping the expanded regions of interest for [C_16_MIM]Br in the absence (red) and in the presence of fluconazole (blue).

The results showed that the chemical shift of H22 did not change, and therefore, it was not influenced by the presence of fluconazole. The chemical shifts of H4-H5, H31, and H11 decreased in the presence of fluconazole. This finding suggests that fluconazole interacts with the IL cationic heads on the surface of the micelles, wherein the ^1^H nuclei of the cationic head are shielded by aromatic or heteroaromatic rings of fluconazole and/or neighboring IL rings [34,35]. Additionally, signals H4 and H5 partially merge in the presence of fluconazole, also indicating the interactions at the micelle surface. The same experiment was performed with [C_16_MIM]Br in the absence and in the presence of fluconazole. The overlapping spectra are shown in Figure 11.

The spectra showed that the chemical shift of H26 increased in the presence of fluconazole. This can be a result of the decrease in the compactness of the IL molecules upon the addition of fluconazole, which can be explained by the break in the hydrophobic interactions between the IL molecules. Therefore, the entry of more water molecules into the aggregate nuclei is facilitated, thereby increasing the polarity inside the aggregates and consequently promoting the deshielding of H26 signals. 

**Figure 12:**
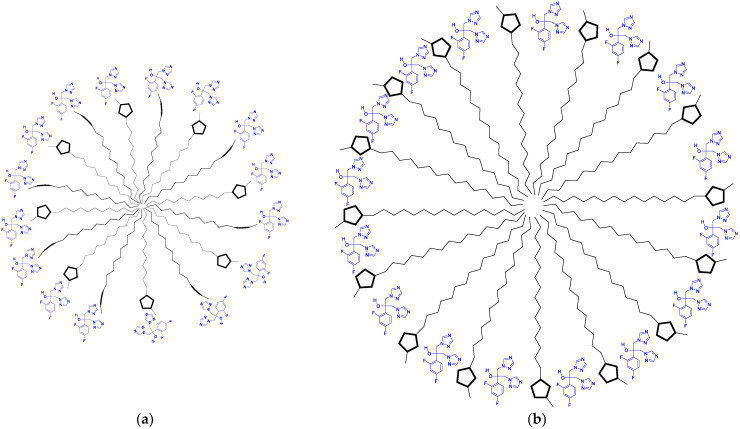
Schematic representation of mixed aggregates formed by (**a**) [C_12_MIM]Br and (**b**) [C_16_MIM]Br with fluconazole.

The chemical shift of H4, H5, H11, and H31 from the IL cationic head also increased, indicating that these nuclei are in the deshielding cone of fluconazole, whereas the opposite behavior was observed for [C_12_MIM]Br. In this case, these IL hydrogen atoms interact with fluconazole via hydrogen bonds, in which the IL molecules are hydrogen donors and the heteroatoms (O and N) from the fluconazole and/or bromide anions are likely to be the hydrogen bond acceptors. This indicates that the fluconazole molecules can be located between the cationic heads in the mixed aggregates. These differences in the interaction mechanism with fluconazole observed for [C_12_MIM]Br and [C_16_MIM]Br are to be expected since the longer alkyl side chain of [C_16_MIM]Br allows for the formation of mixed aggregates that are greater and more compact than those formed by [C_12_MIM]Br. This result is supported by the diffusion data. [C_16_MIM]Br diffuses more slowly in the presence of fluconazole, indicating greater mixed aggregates with stronger intermolecular interactions than [C_12_MIM]Br, which presented higher D values in the presence of fluconazole. Figure 12 presents a schematic representation of the aggregates formed from the ILs with fluconazole. The bromide anions were omitted from the schemes for better visualization. The present work has shown that the size of the alkyl side chain can drive the interactions of ILs with MCT and other active pharmaceutical ingredients.

### 3.3. Antifungal Activity of Emulsion 

In order to have an estimative of the antifungal potential of fluconazole in the emulsion contain ILs, the IC_50_ of emulsions in the presence and absence of fluconazole containing the highest and lowest concentration of IL and prepared using magnetic stirring (MS) and ultrasound irradiation (US 40%) was determined against a susceptible and a resistant *Candida* strain. Results are given in Table 3. 

All emulsions containing ILs showed superior activity when compared only with the FLZ. The most significant difference in activity was obtained with the CG34 strain, probably due to its resistance profile (>64 μg mL^−1^) to FLZ. The HC/US emulsion showed the lowest IC_50_ with the CG34 isolate. Thus, the concentration was 1000 times lower than FLZ alone and statistically lower than emulsions without FLZ. The LC/MS presented the highest IC_50_ compared to the other tested emulsions. The clinical isolate of *C. albicans* showed heterogeneous inhibition. However, emulsions containing FLZ obtained significantly lower concentrations (*p* < 0.05). The LC/US emulsion presented the lowest IC_50_ among the tested emulsions but without a statistical difference compared to the emulsion without the antifungal. On the other hand, some emulsions without FLZ showed no apparent effect compared with FLZ alone. In general, the HC/US emulsion had the best performance and the lowest inhibitory concentrations. On the other hand, the LC/MS emulsion presented the highest IC_50_ for the yeasts tested. The emulsions also showed potent activity in inhibiting a species (*C. glabrata*—CG34) resistant to FLZ. When testing the emulsions without FLZ, we found a pattern: LC and HC showed a more significant effect in the absence of the antifungal for resistant yeast and a diminished effect when fighting sensitive yeast. The emulsions with the best performance were those with C_12_MIM[Br]. Furthermore, concentrations greater than 2.4 mM of the ionic liquid appear to be essential for the isolated activity of white emulsions. Thus, it seems that magnetic stirring provided higher destabilization of the LC emulsion when compared to HC. It is possible that the preparation method explains the difference in activity between the two emulsions in the absence of the antifungal.

### 3.4. *Hen’s Egg Test Chorioallantoic Membrane (HET-CAM)*

**Figure 13:**
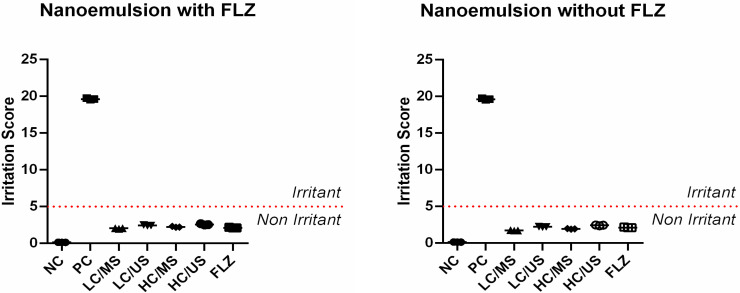
NC: negative control—NaCl (0.9%); PC: positive control—NaOH (0.1 M); FLZ: fluconazole 32 μg mL^−1^; LC/MS: 1.5 mM C_12_MIM[Br]; LC/US: 1.5 mM C_12_MIM[Br]; HC/MS: 3.1 mM C_16_MIM[Br]; HC/US: 3.1 mM C_16_MIM[Br].

The HET-CAM assay showed that the emulsions containing FLZ and those without the antifungal did not present an irritability profile (score < 4.9). In this way, the nanoemulsions were considered to be non-irritating (Figure 13).

All emulsions showed weak irritant potential. The results show that FLZ and ILs did not change the irritability profile of the emulsions. In this way, we can say that emulsions are comparable to the drug alone. The predictive value of the HET-CAM assay is similar to the human skin test [36], with results comparable to the rabbit eye test (Draize test) and with a good correlation with potential skin irritation [37]. In addition, the HET-CAM test is a methodology with high specificity allowing the visualization of non-irritating samples in a fast-response in vivo technique [38].

## 4. Conclusions

This investigation provides a deeper understanding of the stability of [C_12_MIM]Br and [C_16_MIM]Br emulsions. The physical accelerated stability of ILs/fluconazole emulsions, which were prepared using mechanical stirring (MS) and ultrasound (US) at the power of 20% and 40%, were measured at 25 °C and 37 °C using a LUMiSizer. The results showed that the sample prepared using MS had the lowest stability among all the emulsions. These experiments demonstrated the greater stability of [C_16_MIM]Br emulsions, especially when prepared using higher power (US 40%). In contrast, the high-energy method renders less stable [C_12_MIM]Br emulsions. In both cases, creaming destabilization that is characterized by gravitational separation was observed. Additionally, no dependence of stability on the concentration of ILs and temperature was observed. Therefore, a smaller amount of both ILs is sufficient to stabilize the emulsions efficiently, as well as being compatible with applications involving body temperature.

The ^1^H spectra of ILs in the absence and in the presence of MCT indicate that these molecules are inserted into [C_12_MIM]Br hydrophobic micelles, in which the cationic heads probably interact through CH•••π on the aggregate surface. For [C_16_MIM]Br, less compact mixed aggregates were formed with MCT, and the IL cationic heads interacted through hydrogen bonds. In this case, the ^1^H signals splitting suggested that these mixed aggregates are formed by fewer IL monomers, and for this reason, some IL can be left in the solution as free IL micelles. ^1^H DOSY experiments supported that the systems from MCT and [C_12_MIM]Br or [C_16_MIM]Br interacted in a single homogeneous phase. 

Different interaction mechanisms were observed for [C_12_MIM]Br and [C_16_MIM]Br in the presence of fluconazole. [C_16_MIM]Br formed larger and more compact mixed aggregates with fluconazole inserted between the IL cationic heads. In the mixed aggregates formed from [C_12_MIM]Br, the fluconazole molecules were on the aggregate surface. This result is supported by diffusion data, in which [C_16_MIM]Br diffuses slowly in the presence of fluconazole; this indicates stronger intermolecular interactions than those in [C_12_MIM]Br.

A preliminary antifungal assay showed that emulsions containing ILs showed superior activity when compared only with the FLZ. The HC/US emulsion had the best performance and the LC/MS emulsion the worse for the yeasts tested. The emulsions also showed potent activity in inhibiting a species (*C. glabrata*—CG34) resistant to FLZ. The preparation method can possibly explain the difference in the antifungal activity of emulsions. More assays need be performed to confirm this hypothesis. Finally, it is worth noting that all emulsions showed weak irritant potential in the HET-CAM assay. 

## Figures and Tables

**Table 1:** Diffusion coefficient (D × 10^−13^ m^2^ s^−1^) of [C_12_MIM]Br and [C_16_MIM]Br and their mixtures with MCT.

	D_H11_	D_H22_	D_H31_	Average (±SD)
[C_12_MIM]Br	8.47	8.68	8.39	8.51 (±0.12)
[C_12_MIM]Br + 0.5 mL MCT	7.86	7.85	7.59	7.77 (±0.12)
[C_12_MIM]Br + 0.10 mL MCT	7.78	7.63	6.96	7.46 (±0.36)
[C_12_MIM]Br + 0.15 mL MCT	6.86	- ^a^	6.46	5.77 (±0.20) ^b^
	D_H11_	D_H26_	D_H31_	Average (±SD)
[C_16_MIM]Br	- ^a^	7.94	7.10	7.52 (±0.42) ^b^
[C_16_MIM]Br + 0.5 mL MCT	6.46	5.91	5.88	6.08 (±0.27)
[C_16_MIM]Br + 0.10 mL MCT	5.25	5.55	5.69	5.50 (±0.18)
[C_16_MIM]Br + 0.15 mL MCT	- ^a^	- ^a^	4.84	-

^a^ A lack of correlation of the curve points was observed, and the determination of D was compromised. ^b^ Average values calculated in duplicate.

**Table 2:** Diffusion coefficient (D × 10^−13^ m^2^ s^−1^) for [C_12_MIM]Br and [C_16_MIM]Br, and their mixtures with fluconazole.

	D_H11_	D_H22_	D_H31_	D_H4,H5_	Average (±SD)
[C_12_MIM]Br	9.06	9.04	8.89	8.81	8.95 (±0.10)
[C_12_MIM]Br + fluconazole	8.14	8.72	7.67	8.96	8.37 (±0.50)
	D_H11_	D_H26_	D_H31_	D_H4.H5_	Average (±SD)
[C_16_MIM]Br	5.13	4.96	4.72	6.06	5.22 (±0.51)
[C_16_MIM]Br + fluconazole	4.97	5.78	5.45	4.62	5.21 (±0.44)

**Table 3:** Antifungal activity (IC_50_ in nM) of emulsion containing ILs prepared in the ultrasound and magnetic stirring (MS).

C_12_MIMBr					C_16_MIMBr			
	CAMS1		CG34		CAMS1		CG34	
IL conc. ^a^/ prep. Mode	Pres. Fluc. ^b^	Abs. Fluc. ^c^	Pres. Fluc. ^b^	Abs. Fluc. ^c^	Pres. Fluc. ^b^	Abs. Fluc. ^c^	Pres. Fluc. ^b^	Abs. Fluc. ^b^
LC/MS	0.642	0.704	0.573	0.948	0.363	0.761	2.727	1.623
LC/US	0.568	0.550	0.929	1.636	0.327	0.659	1.749	0.760
HC^/^MS	0.974	2.683	0.433	0.326	0.894	1.537	0.193	0.540
HC/US	1.218	3.301	0.383	0.331	0.559	0.928	0.187	0.416

^a^ HC = highest concentration of IL (3.6 for C_12_MIMBr and 3.1 for C_16_MIMBr) and LC = lowest concentration of IL (1.5 for C_12_MIMBr and 1.3 for C_16_MIMBr). ^b^ Presence (pres. Fluc.) and ^c^ absence of fluconazole (Abs. Fluc.) were both compared with the same microdilution, so even in the absence of fluconazole the concentration shown was that of the dilution achieved. Fluconazole IC_50_ for CAMS1 (1.010 nM) and CG34 (0.209 nM).

## Data Availability

Not applicable.

## Supplementary Materials

The following are available online at https://www.mdpi.com/article/10.3390/pharmaceutics14040710/s1, Table S1: Instability Index of emulsions at 25 °C, Table S2: Instability Index of emulsions at 37 °C, Figures S1–S30: Transmission profile as a function of position in the cuvette of the emulsions, Figure S31: ^1^H NMR for C_12_MIM[Br] in D_2_O at 25 °C, Figure S32: ^1^H NMR for C_16_MIM[Br] in D_2_O at 25 °C, Figure S33: Images of emulsions (a) C_12_MIM[Br] + 0.15 mL of MCT; e (b) C_16_MIM[Br] + 0.15 mL of MCT; em D_2_O, Figure S34: DOSY graph for (a) C_12_MIM[Br] − H22; (b) C_12_MIM[Br] + 0.05 mL of MCT − H22; (c) C_16_MIM[Br] − H26; (d) C_16_MIM[Br] + 0.05 mL of MCT − H26, Figure S35: DOSY graph for (a) C_12_MIM[Br] − H22; (b) C_12_MIM[Br] + fluconazole − H22; (c) C_16_MIM[Br] – H26; (d) C_16_MIM[Br] + fluconazole − H26.
