# Supplementary material for: Antifungal Activity and Stability of Fluconazole Emulsion Containing Ionic Liquids Explained by Intermolecular Interactions
Source: Pharmaceutics. 2022 Mar 26;14(4):710. doi: 10.3390/pharmaceutics14040710 (PMC9026797; doi:10.3390/pharmaceutics14040710)
Supplement: Supplementary file 1 [file pharmaceutics-14-00710-s001.zip › pharmaceutics-1639298-supplementary.pdf]

Supplementary Materials: Antifungal Activity and Stability of Fluconazole Emulsion Containing Ionic Liquids Explained by Intermolecular Interactions

Bruno L. Hennemann, Caroline R. Bender, Guilherme S. Moleta, Ânderson R. Carvalho, Luana C. G. Bazana, Alexandre M. Fuentefria and Clarissa P. Frizzo

1. Instability Index.

Table S1. Instability Index of emulsions at 25 °C.

	**1.5 mM**	**2.4 mM**	**3.6 mM**	**1.3 mM**	**2.06 mM**	**3.1 mM**
	**C_{12}MIM[Br]**	**C_{12}MIM[Br]**	**C_{12}MIM[Br]**	**C_{16}MIM[Br]**	**C_{16}MIM[Br]**	**C_{16}MIM[Br]**
**MS**	0.089	0.095	0.396	0.856	0.865	0.886
**US 20%**	0.329	0.64	0.666	0.83	0.834	0.865
**US 40%**	0.613	0.637	0.664	0.799	0.751	0.763

Table S2. Instability Index of emulsions at 37 °C.

	**1.5 mM**	**2.4 mM**	**3.6 mM**	**1.3 mM**	**2.06 mM**	**3.1 mM**
	**C_{12}MIM[Br]**	**C_{12}MIM[Br]**	**C_{12}MIM[Br]**	**C_{16}MIM[Br]**	**C_{16}MIM[Br]**	**C_{16}MIM[Br]**
**MS**	0.158	0.104	0.248	0.88	0.876	0.886
**US 20%**	0.32	0.582	0.663	0.845	0.818	0.858
**US 40%**	0.552	0.608	0.599	0.699	0.676	0.741

2. Transmission Profiles.

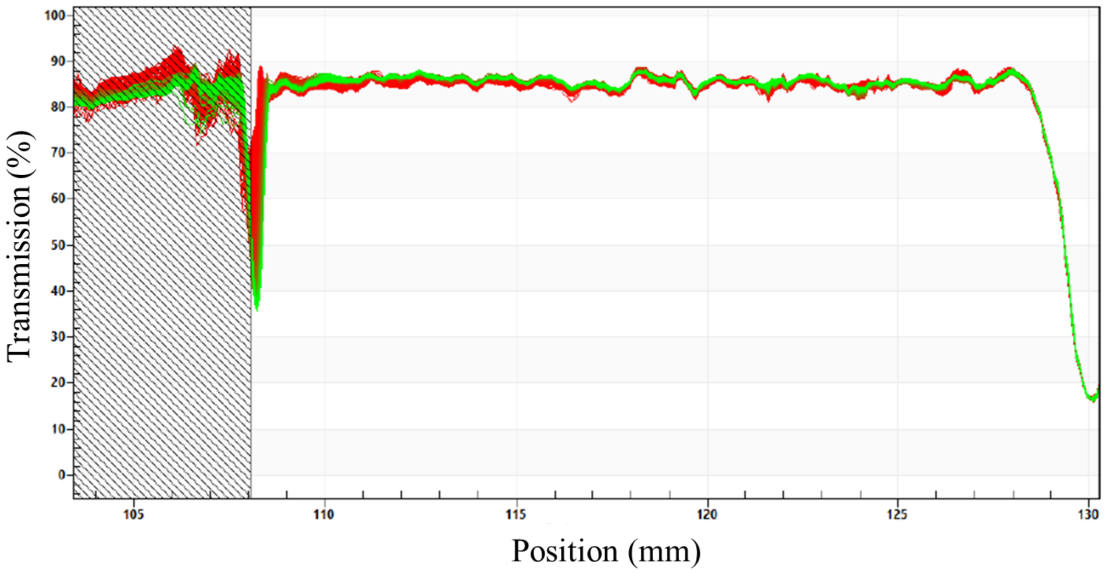

Figure S1. Transmission profile as a function of position in the cuvette at 25 °C of the emulsion with 1.5 mM LI C_{12}MIM[Br] prepared by MS.

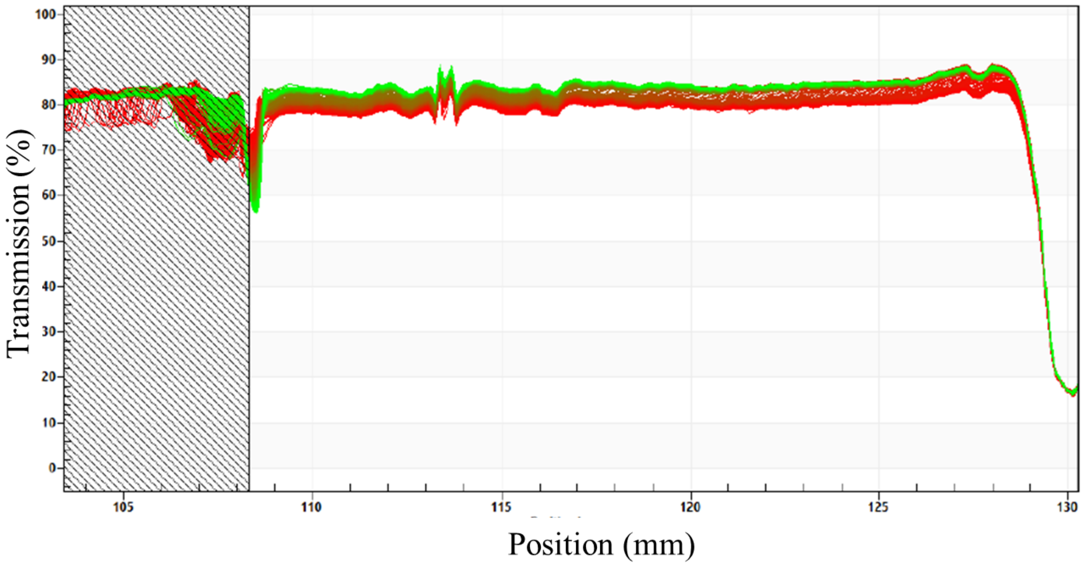

Figure S2. Transmission profile as a function of position in the cuvette at 25 °C of the emulsion with 1.5 mM LI C_{12}MIM[Br] prepared by US 20%.

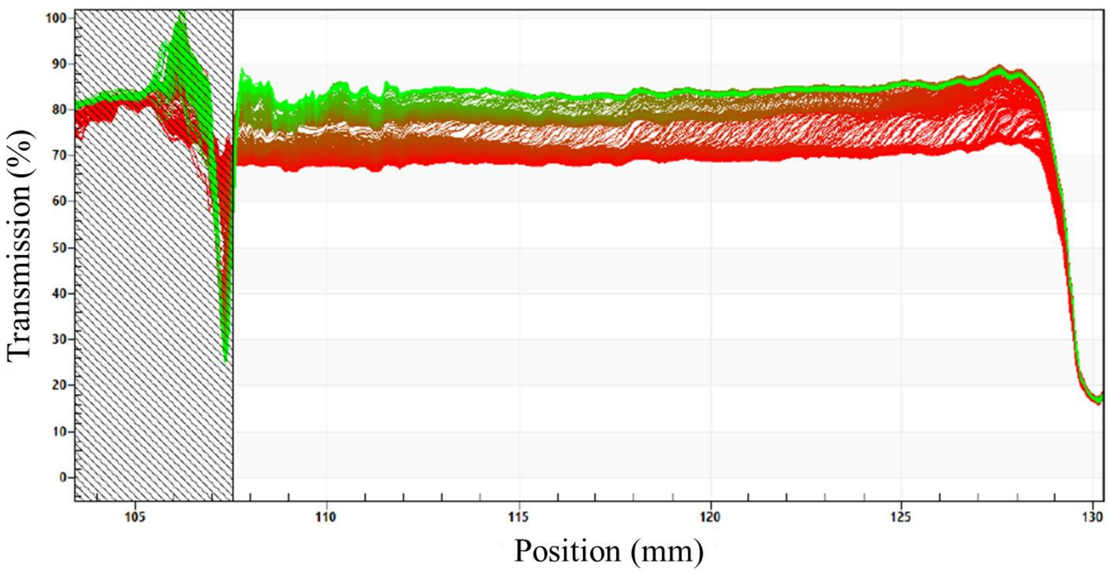

Figure S3. Transmission profile as a function of position in the cuvette at 25 °C of the emulsion with 1.5 mM LI $C_{12}MIM[Br]$ prepared by US 40%.

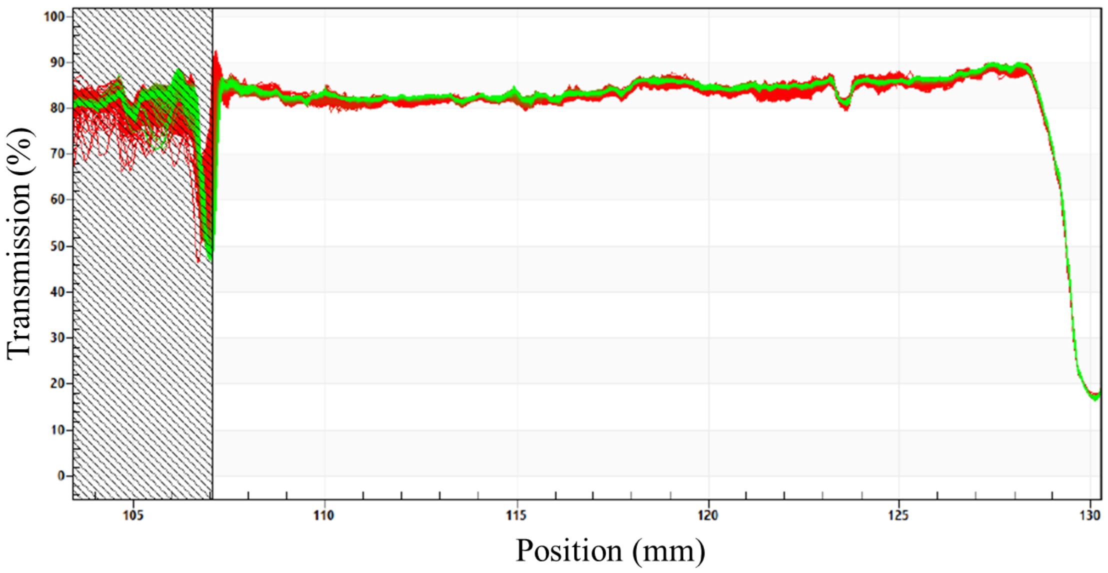

Figure S4. Transmission profile as a function of position in the cuvette at 25 °C of the emulsion with 2.4 mM LI $C_{12}MIM[Br]$ prepared by MS.

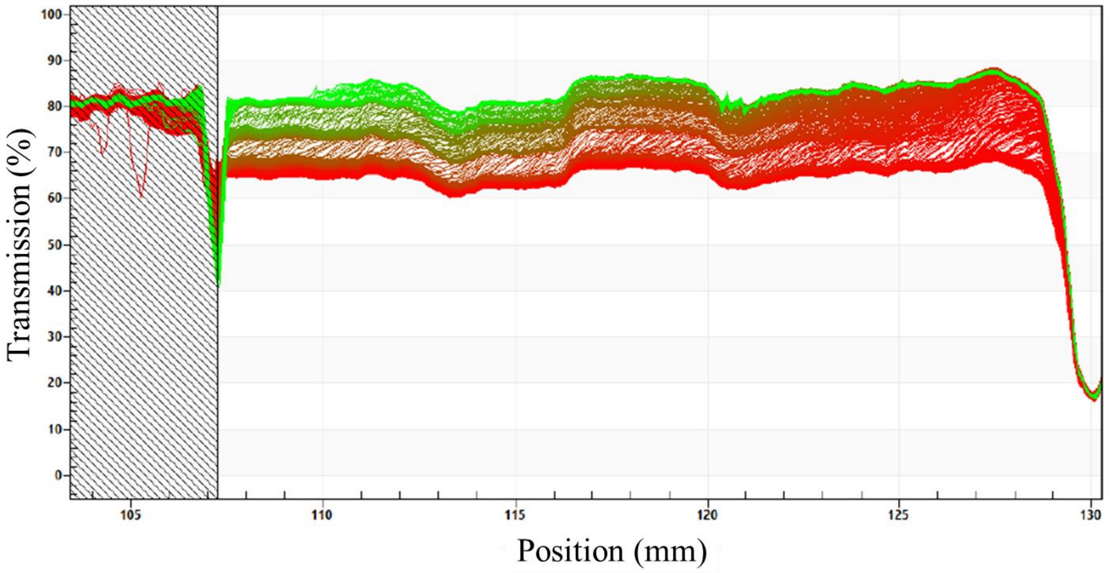

Figure S5. Transmission profile as a function of position in the cuvette at 25 °C of the emulsion with 2.4 mM LI C_{12}MIM[Br] prepared by US 20%.

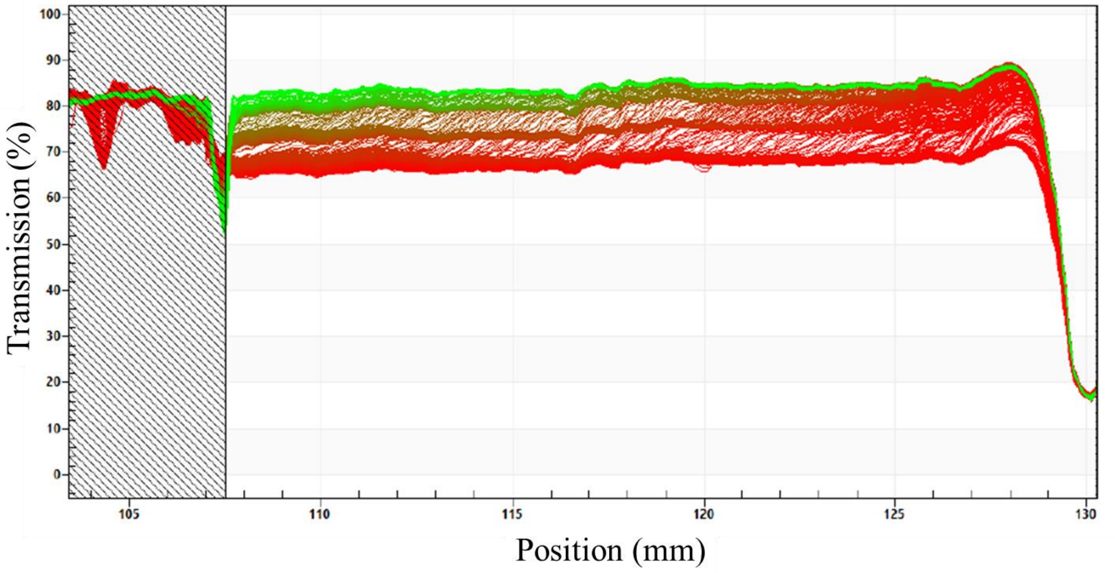

Figure S6. Transmission profile as a function of position in the cuvette at 25 °C of the emulsion with 2.4 mM LI C_{12}MIM[Br] prepared by US 40%.

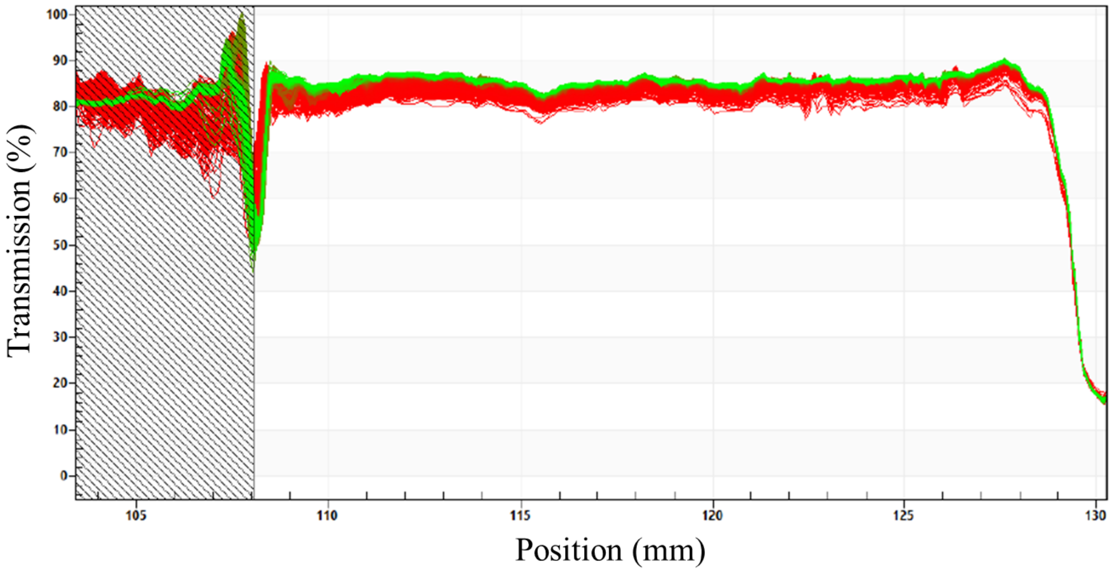

Figure S7. Transmission profile as a function of position in the cuvette at 25 °C of the emulsion with 3.6 mM LI C_{12}MIM[Br] prepared by MS.

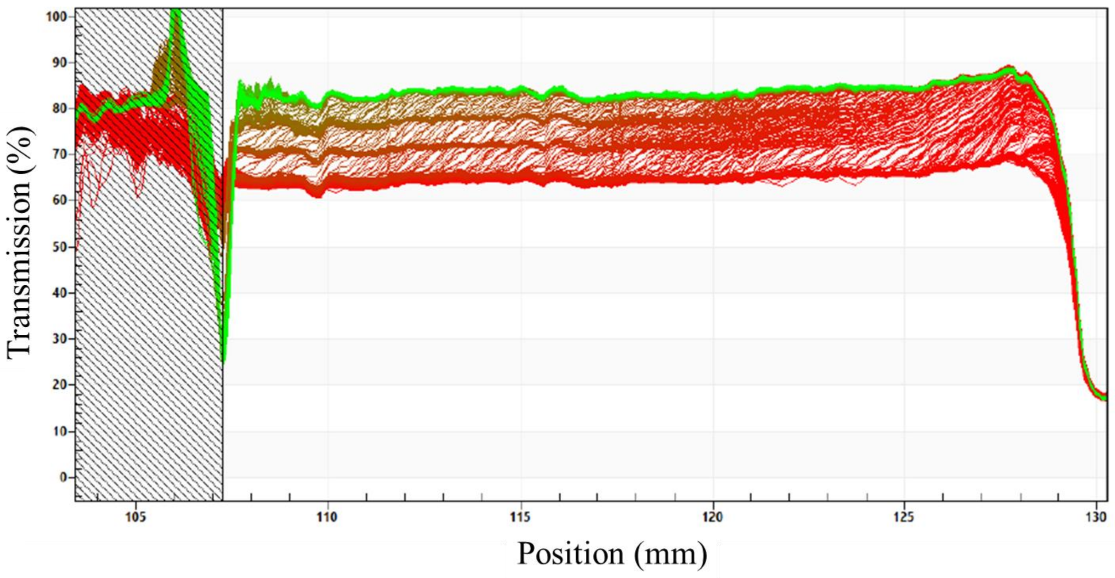

Figure S8. Transmission profile as a function of position in the cuvette at 25 °C of the emulsion with 3.6 mM LI C_{12}MIM[Br] prepared by US 20%.

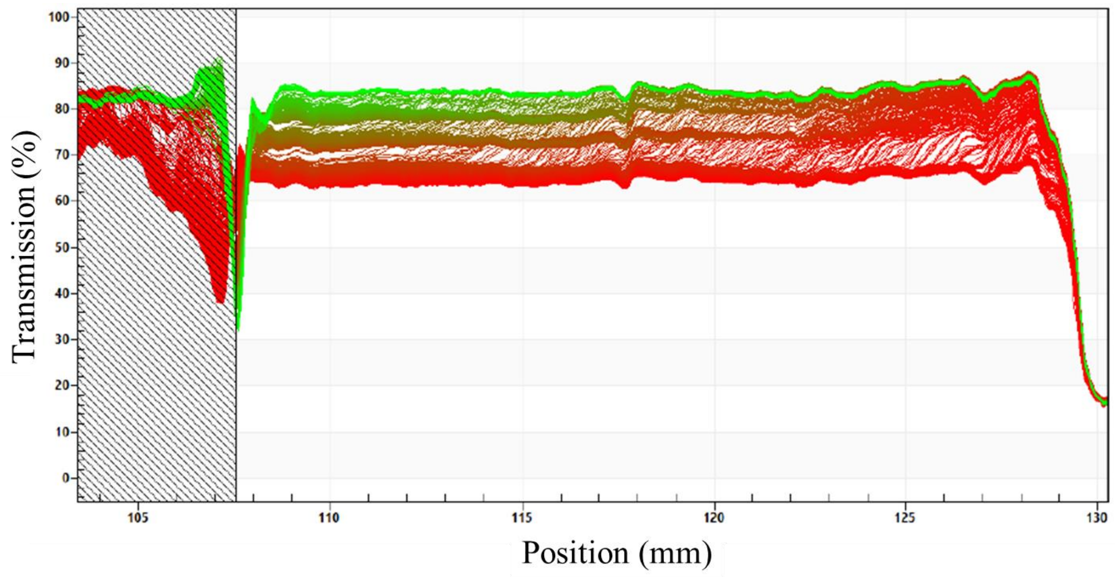

Figure S9. Transmission profile as a function of position in the cuvette at 25 ^{O}C of the emulsion with 3.6 mM LI $C_{12}MIM[Br]$ prepared by US 40%.

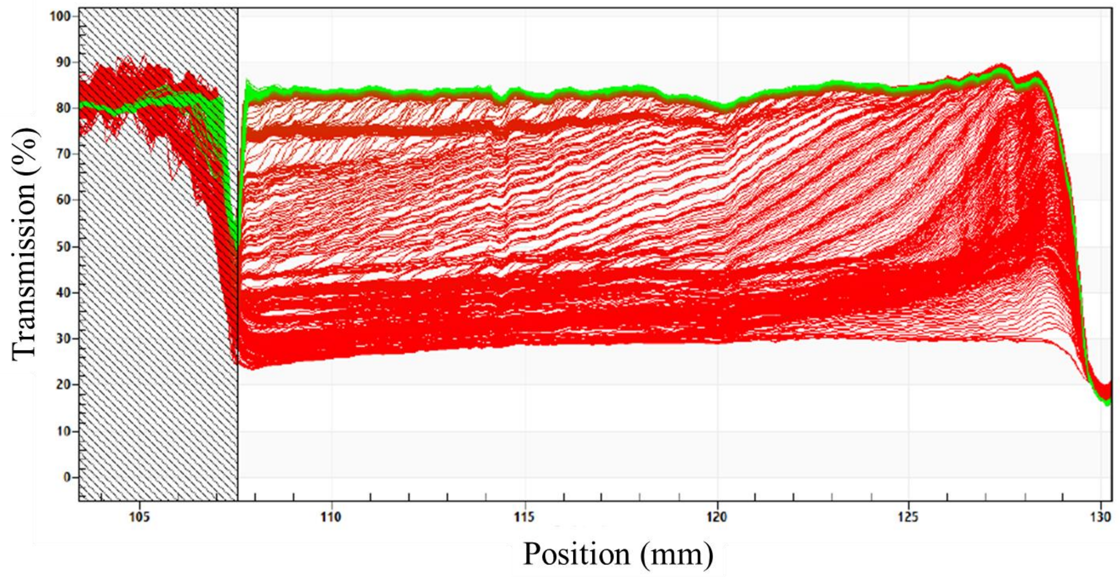

Figure S10. Transmission profile as a function of position in the cuvette at 25 ^{O}C of the emulsion with 1.3 mM LI $C_{16}MIM[Br]$ prepared by MS.

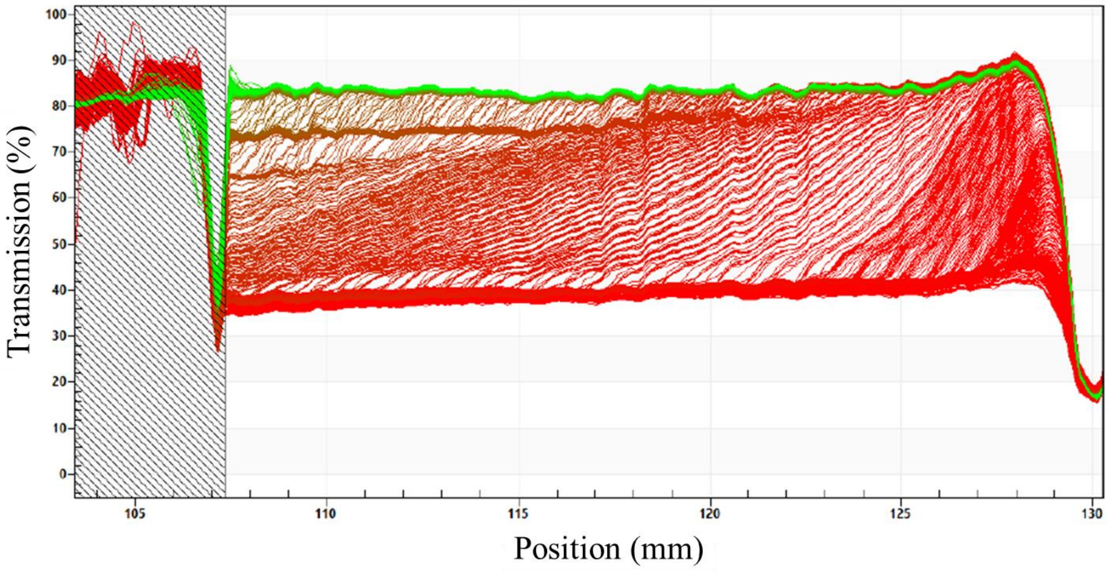

Figure S11. Transmission profile as a function of position in the cuvette at 25 ^{O}C of the emulsion with 1.3 mM LI $C_{16}MIM[Br]$ prepared by US 20%.

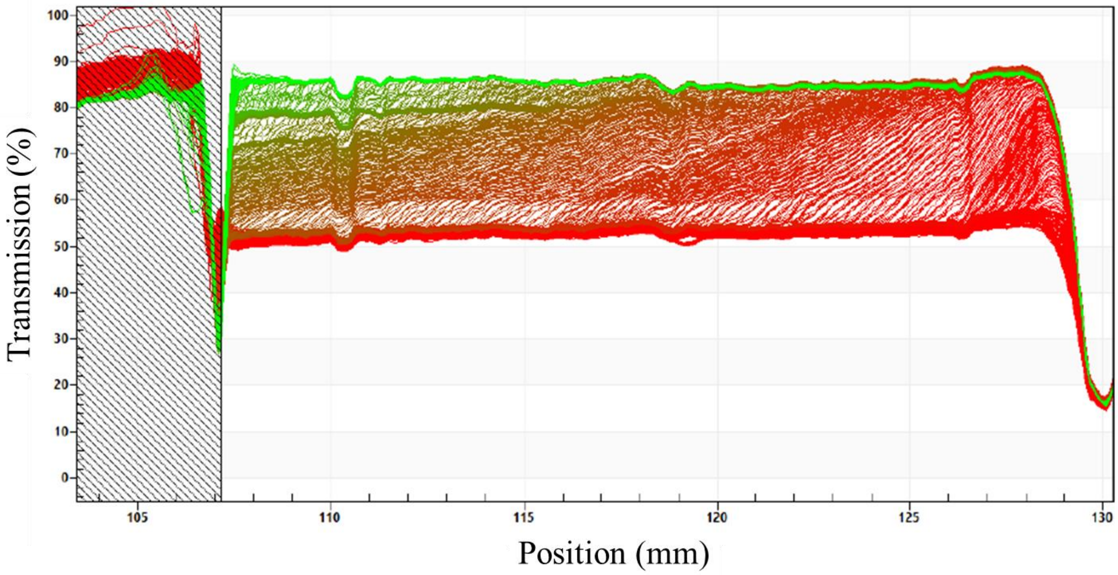

Figure S12. Transmission profile as a function of position in the cuvette at 25 ^{O}C of the emulsion with 1.3 mM LI $C_{16}MIM[Br]$ prepared by US 40%.

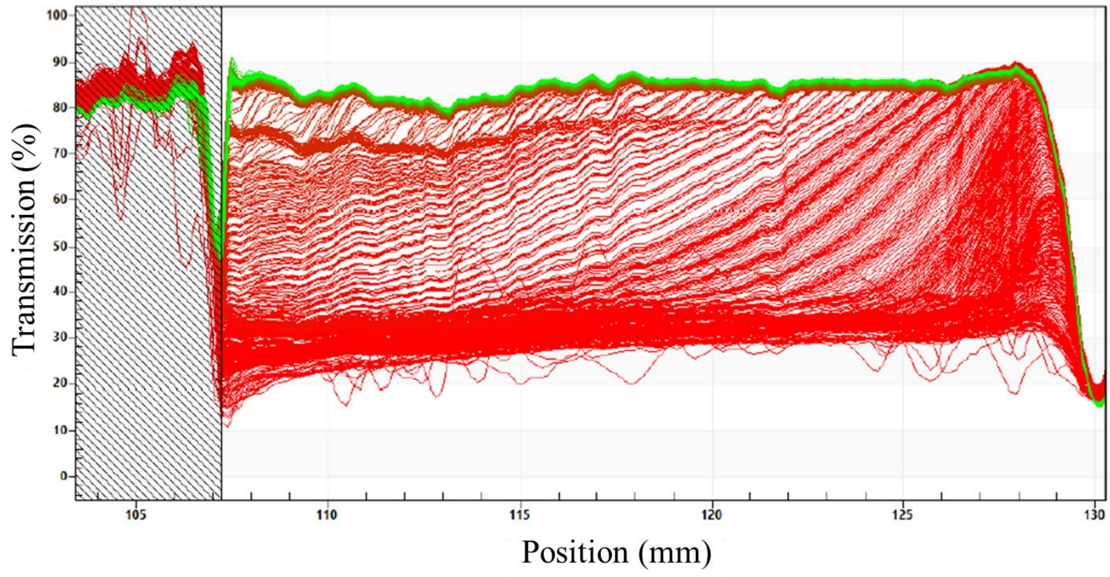

Figure S13. Transmission profile as a function of position in the cuvette at 25 ^{O}C of the emulsion with 2.06 mM LI $C_{16}MIM[Br]$ prepared by MS.

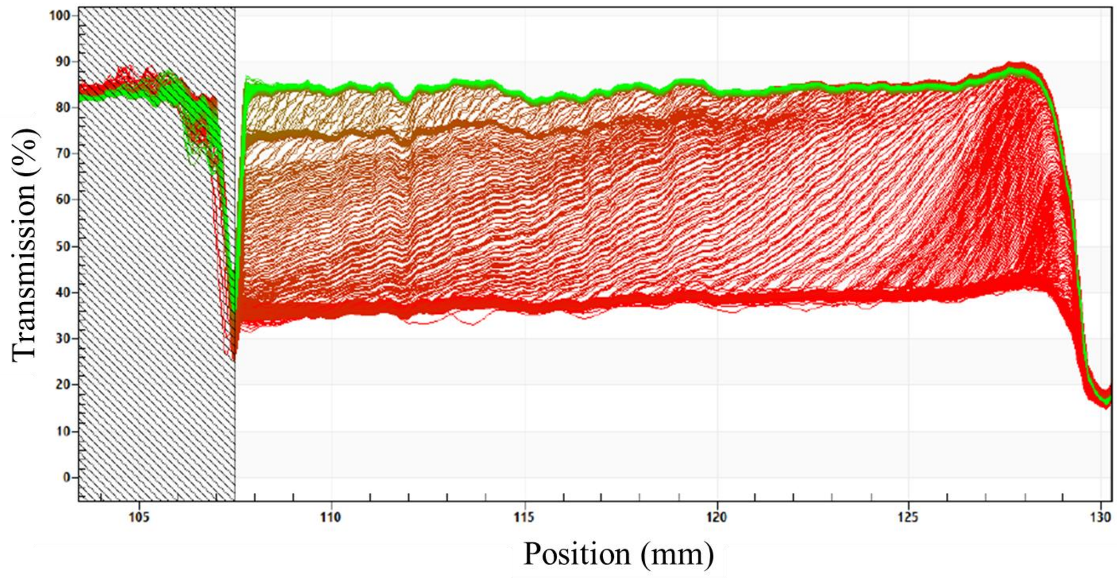

Figure S14. Transmission profile as a function of position in the cuvette at 25 ^{O}C of the emulsion with 2.06 mM LI $C_{16}MIM[Br]$ prepared by US 20%.

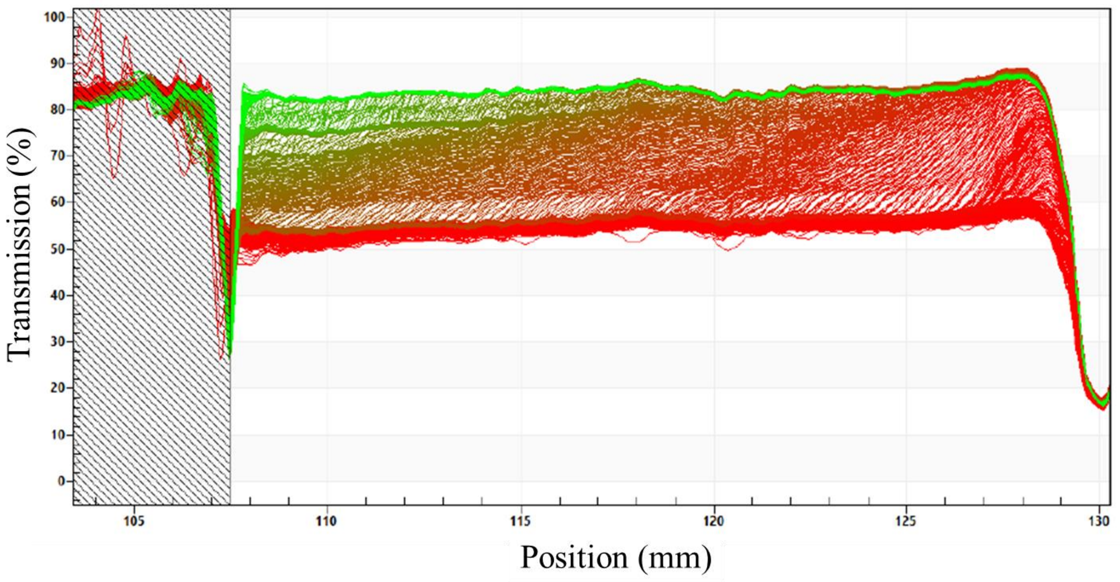

Figure S15. Transmission profile as a function of position in the cuvette at 25 °C of the emulsion with 2.06 mM LI C_{16}MIM[Br] prepared by US 40%.

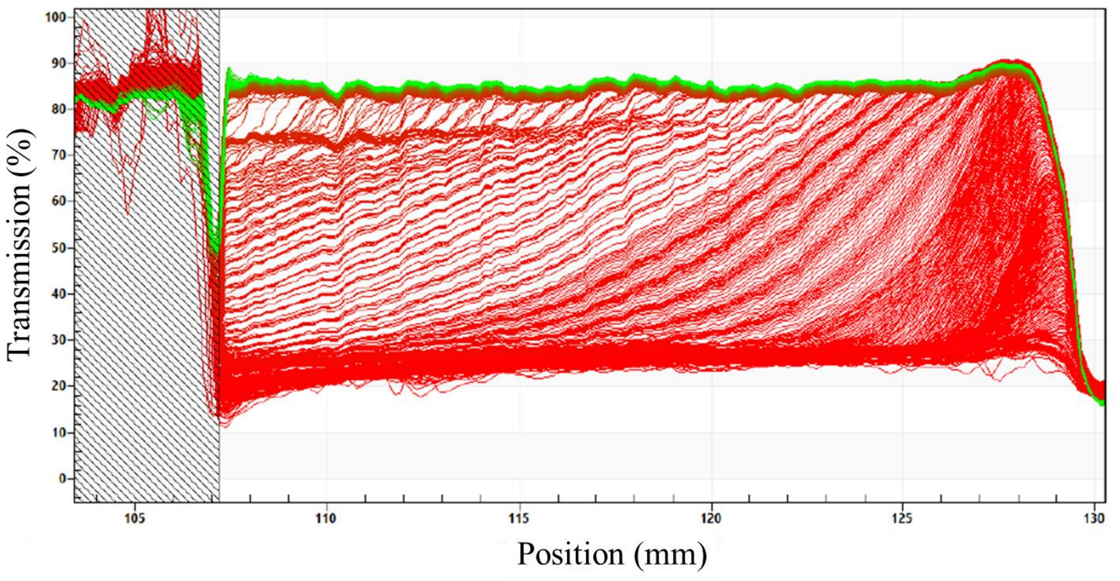

Figure S16. Transmission profile as a function of position in the cuvette at 25 °C of the emulsion with 3.1 mM LI C_{16}MIM[Br] prepared by MS.

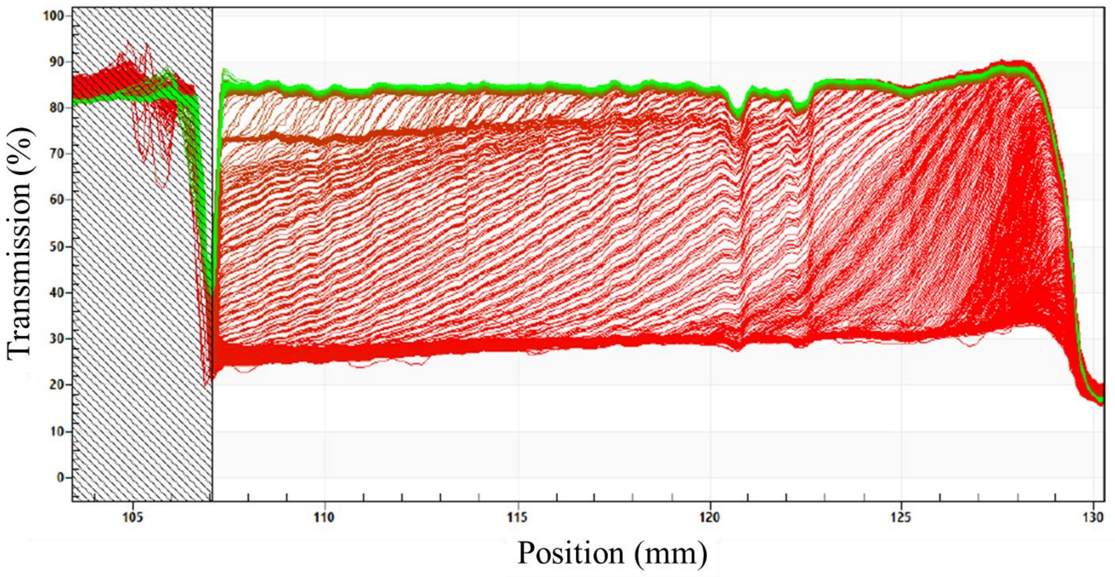

Figure S17. Transmission profile as a function of position in the cuvette at 25 ^{O}C of the emulsion with 3.1 mM LI $C_{16}MIM[Br]$ prepared by US 20%.

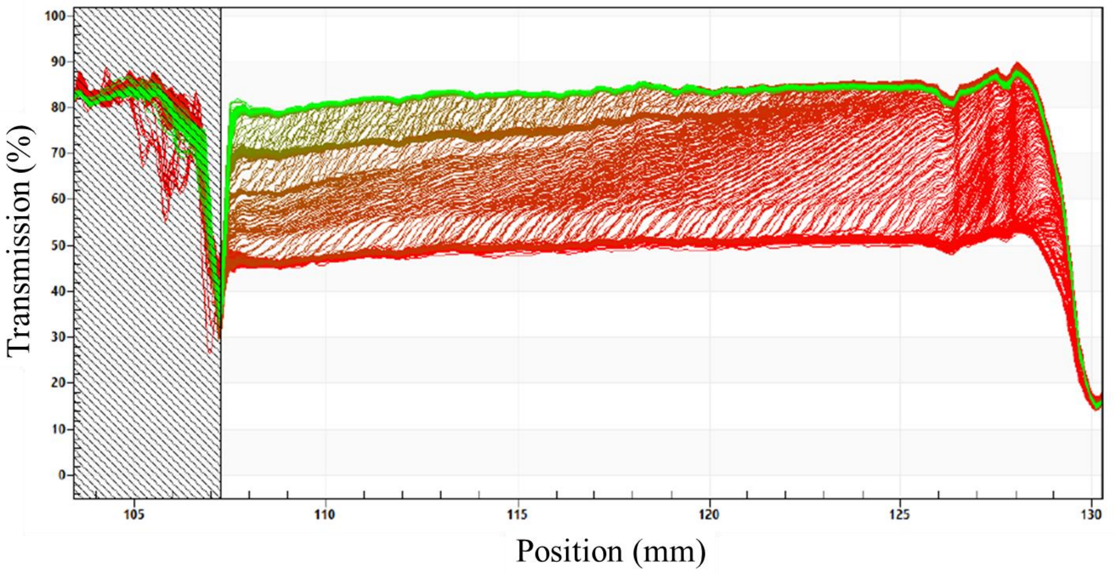

Figure S18. Transmission profile as a function of position in the cuvette at 25 ^{O}C of the emulsion with 3.1 mM LI $C_{16}MIM[Br]$ prepared by US 40%.

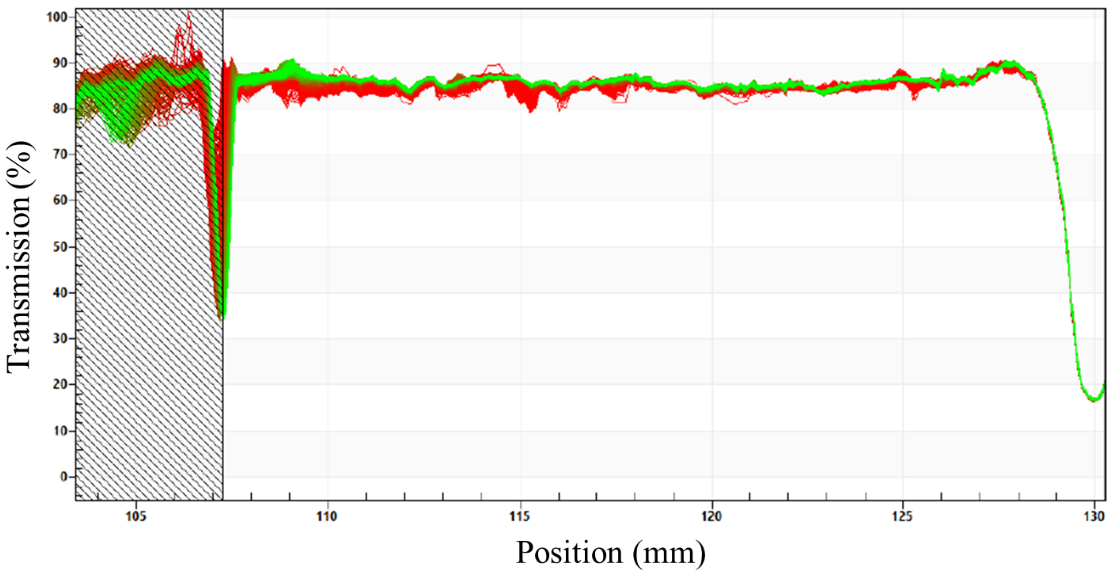

Figure S19. Transmission profile as a function of position in the cuvette at 37 °C of the emulsion with 1.5 mM LI $C_{12}MIM[Br]$ prepared by MS.

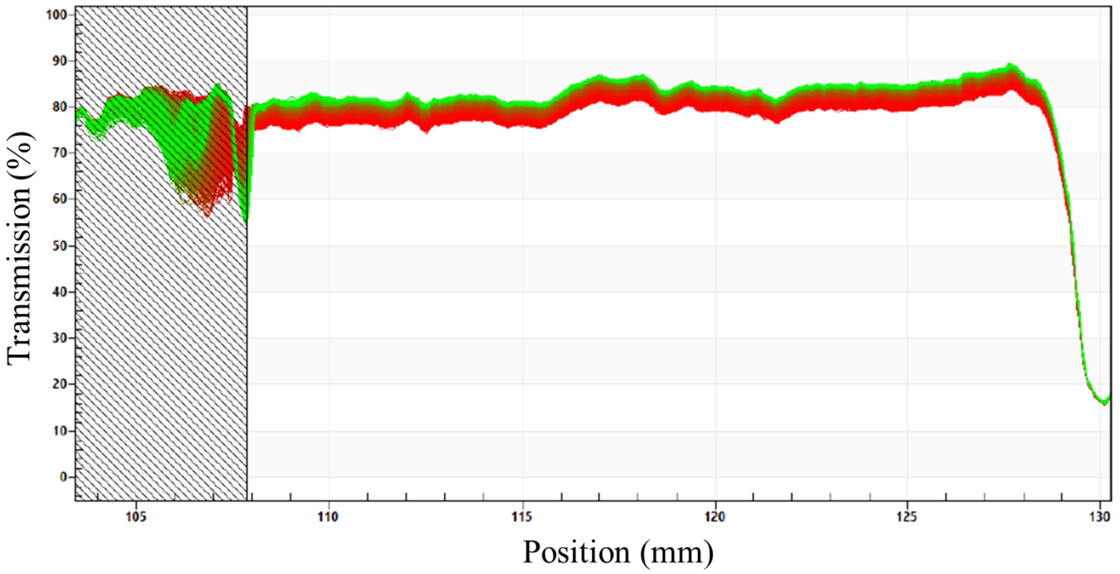

Figure S20. Transmission profile as a function of position in the cuvette at 37 °C of the emulsion with 1.5 mM LI $C_{12}MIM[Br]$ prepared by US 20%.

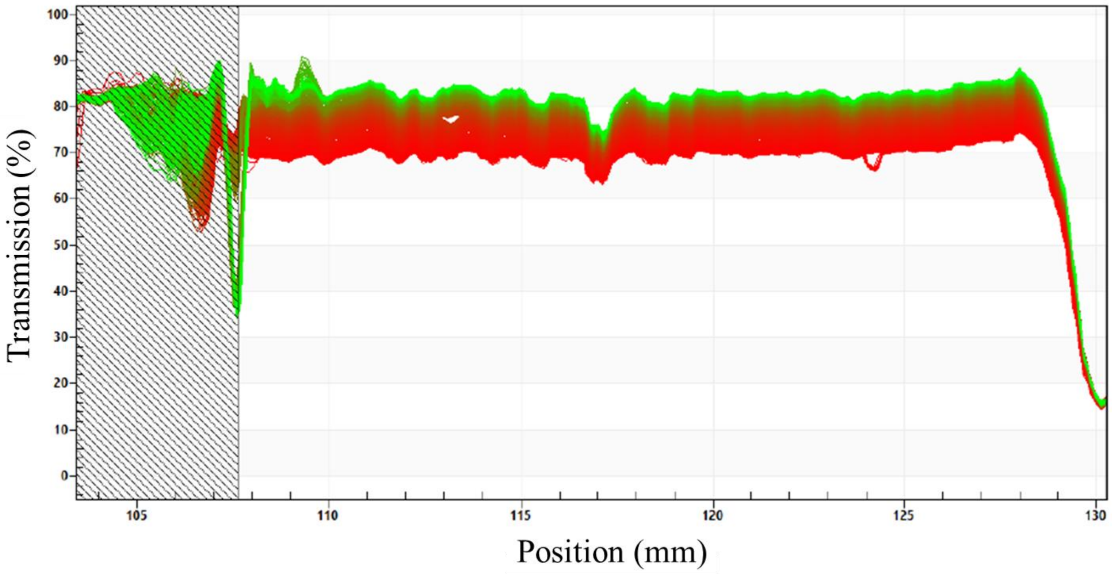

Figure S21. Transmission profile as a function of position in the cuvette at 37 ^{o}C of the emulsion with 1.5 mM LI $C_{12}MIM[Br]$ prepared by US 40%.

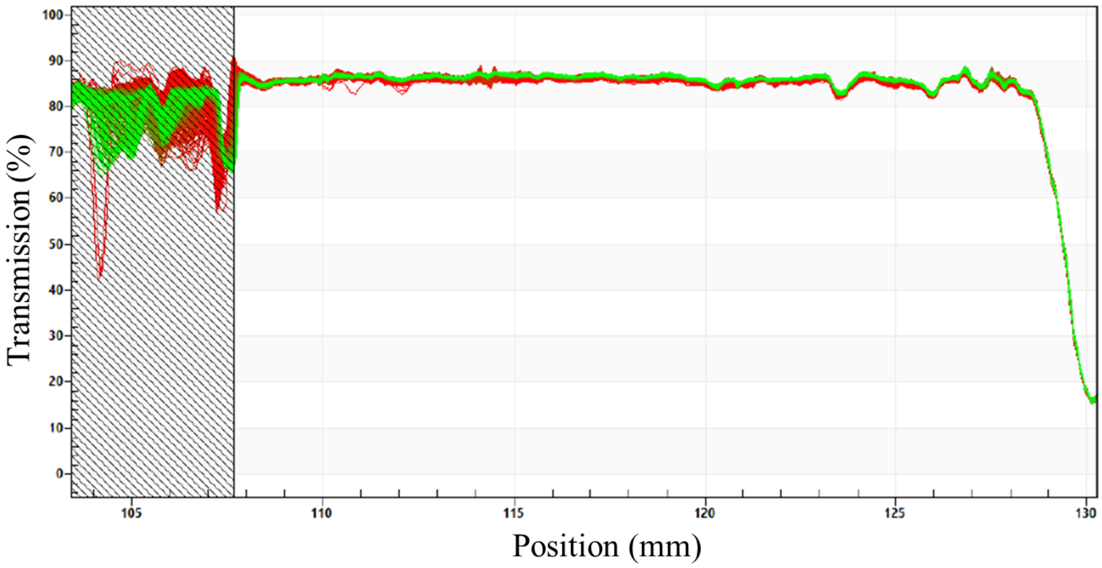

Figure S22. Transmission profile as a function of position in the cuvette at 37 ^{o}C of the emulsion with 2.4 mM LI $C_{12}MIM[Br]$ prepared by MS.

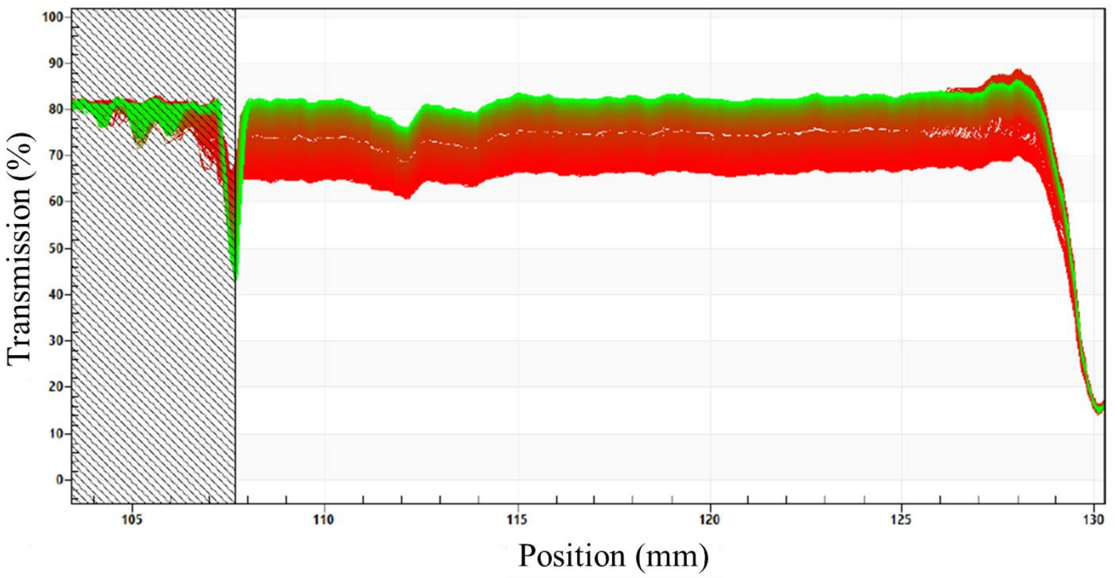

Figure S23. Transmission profile as a function of position in the cuvette at 37 ^{O}C of the emulsion with 2.4 mM LI C_{12}MIM[Br] prepared by US 20%.

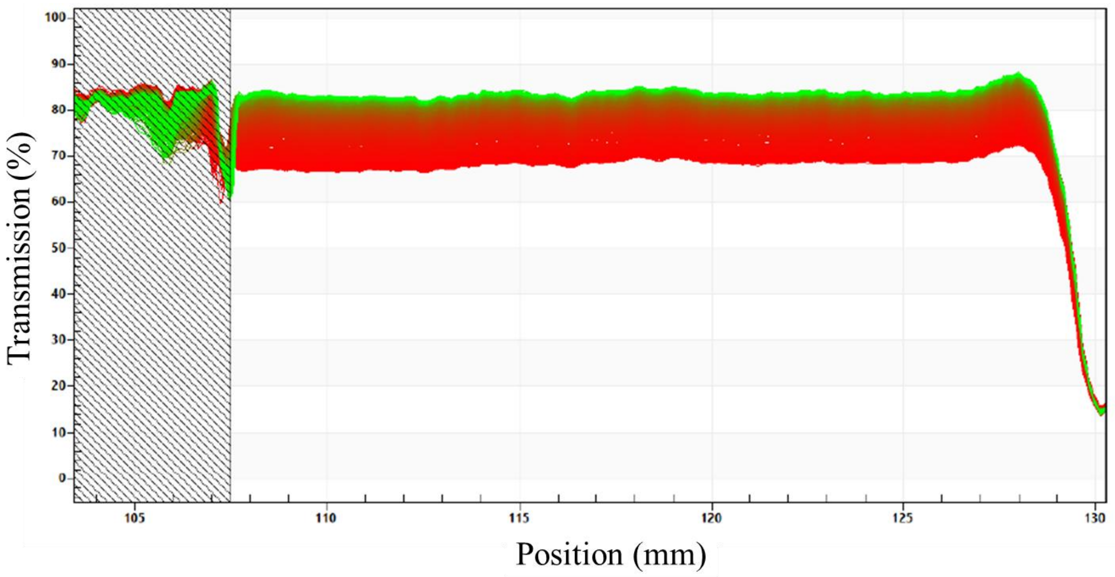

Figure S24. Transmission profile as a function of position in the cuvette at 37 ^{O}C of the emulsion with 2.4 mM LI C_{12}MIM[Br] prepared by US 40%.

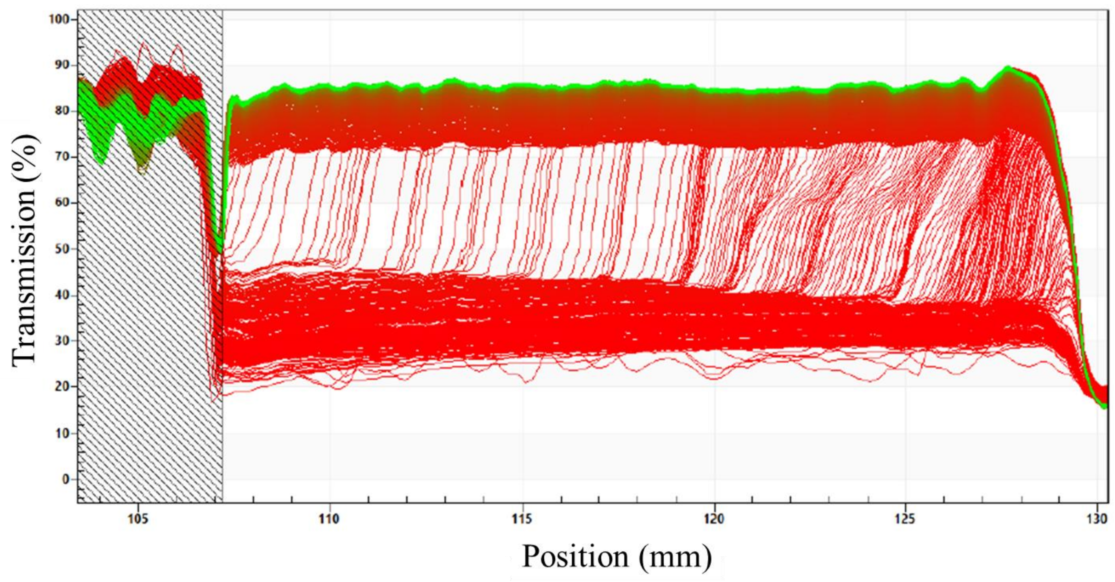

Figure S25. Transmission profile as a function of position in the cuvette at 37 ^{O}C of the emulsion with 2.06 mM LI $C_{16}MIM[Br]$ prepared by MS.

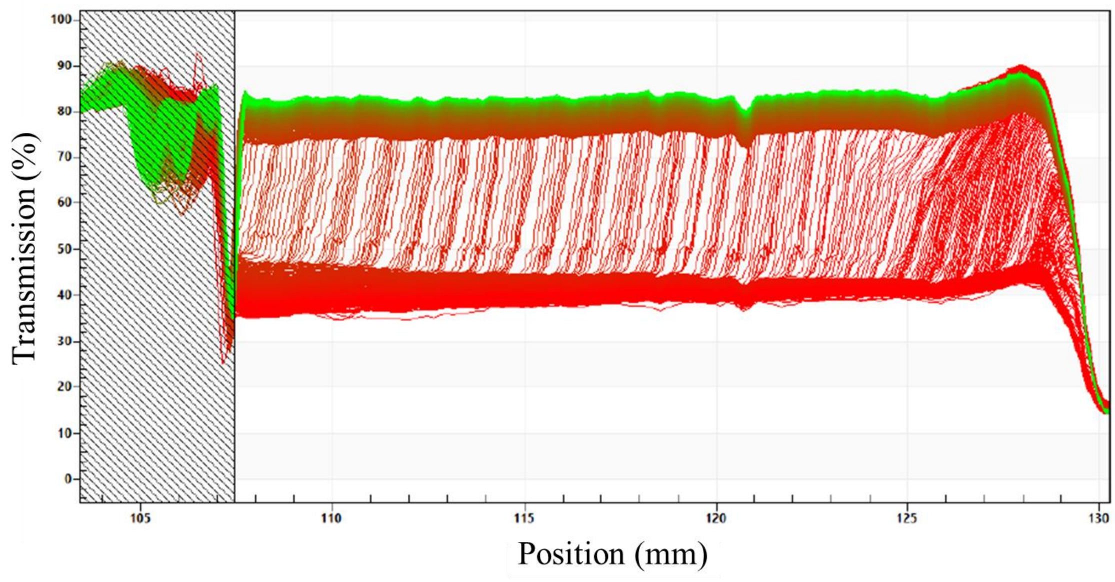

Figure S26. Transmission profile as a function of position in the cuvette at 37 ^{O}C of the emulsion with 2.06 mM LI $C_{16}MIM[Br]$ prepared by US 20%.

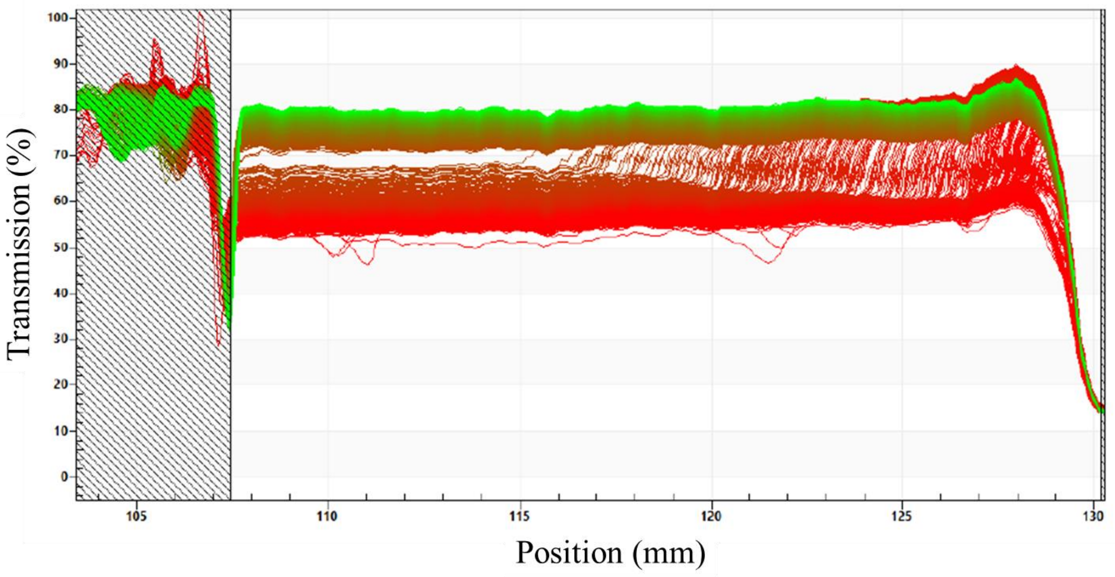

Figure S27. Transmission profile as a function of position in the cuvette at 37 ^{O}C of the emulsion with 2.06 mM LI C_{16}MIM[Br] prepared by US 40%.

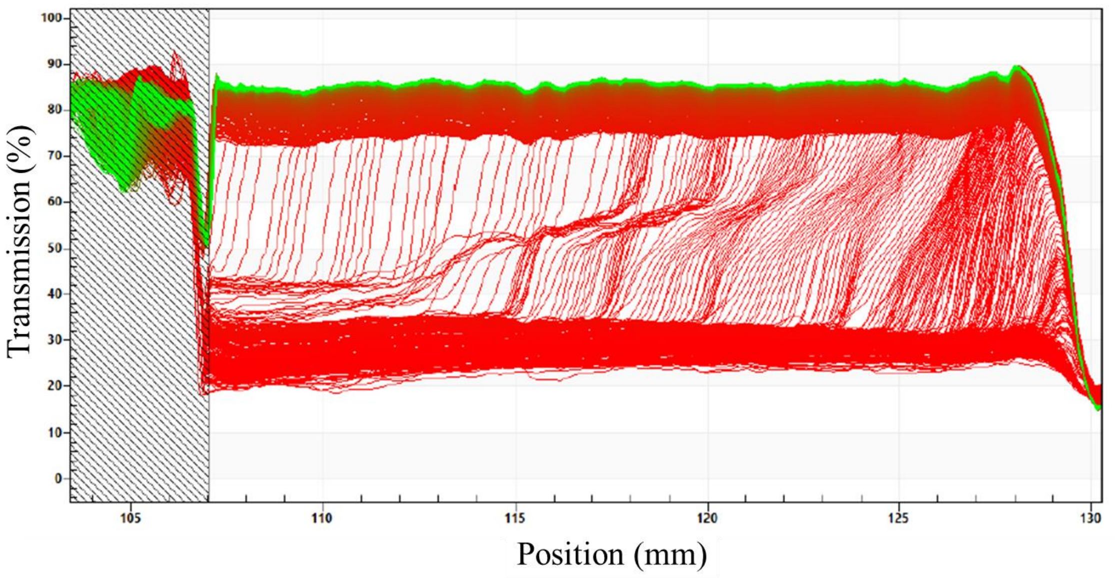

Figure S28. Transmission profile as a function of position in the cuvette at 37 ^{O}C of the emulsion with 3.1 mM LI C_{16}MIM[Br] prepared by MS.

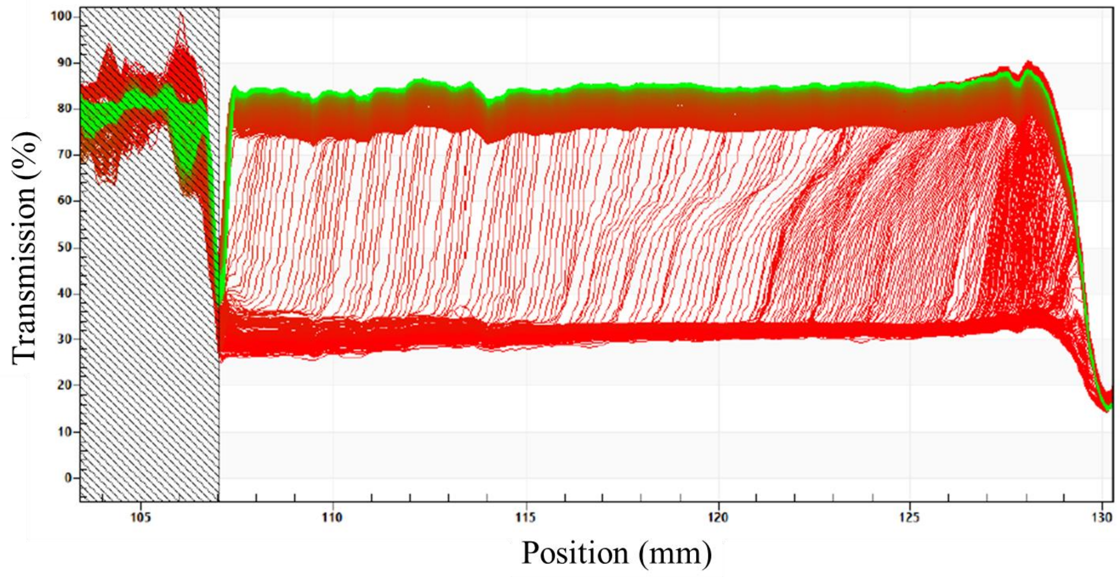

Figure S29. Transmission profile as a function of position in the cuvette at 37 °C of the emulsion with 3.1 mM LI C_{16}MIM[Br] prepared by US 20%.

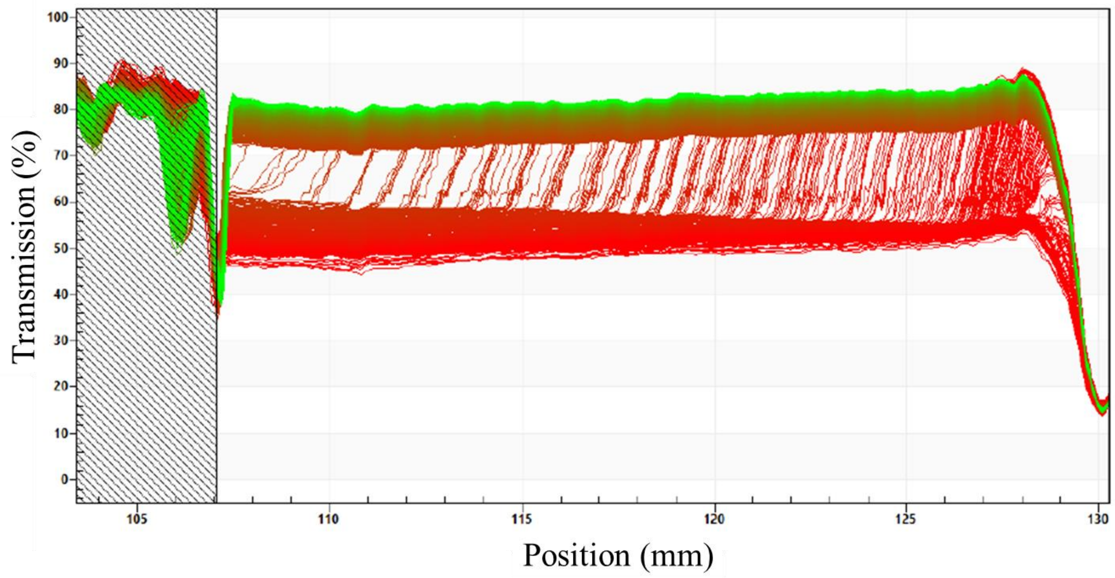

Figure S30. Transmission profile as a function of position in the cuvette at 37 °C of the emulsion with 3.1 mM LI C_{16}MIM[Br] prepared by US 40%.

3. ^{1}H NMR spectra of pure ILs.

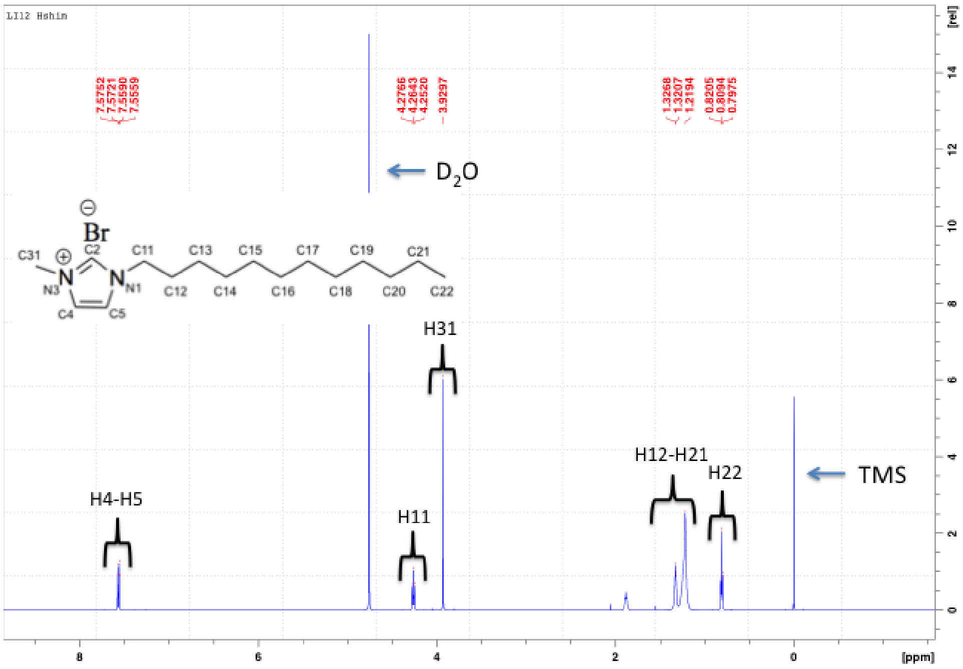

Figure S31. ^{1}H NMR for C_{12}MIM[Br] in D_2O at 25 °C.

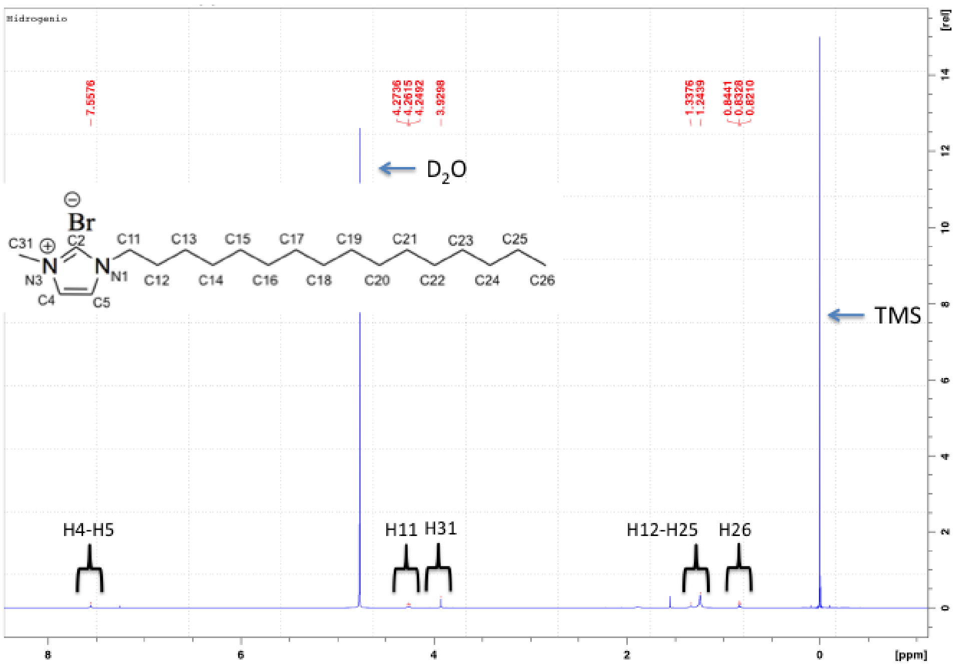

Figure S32. ^{1}H NMR for C_{16}MIM[Br] in D_2O at 25 °C.

4. DOSY graphics for the mixes.

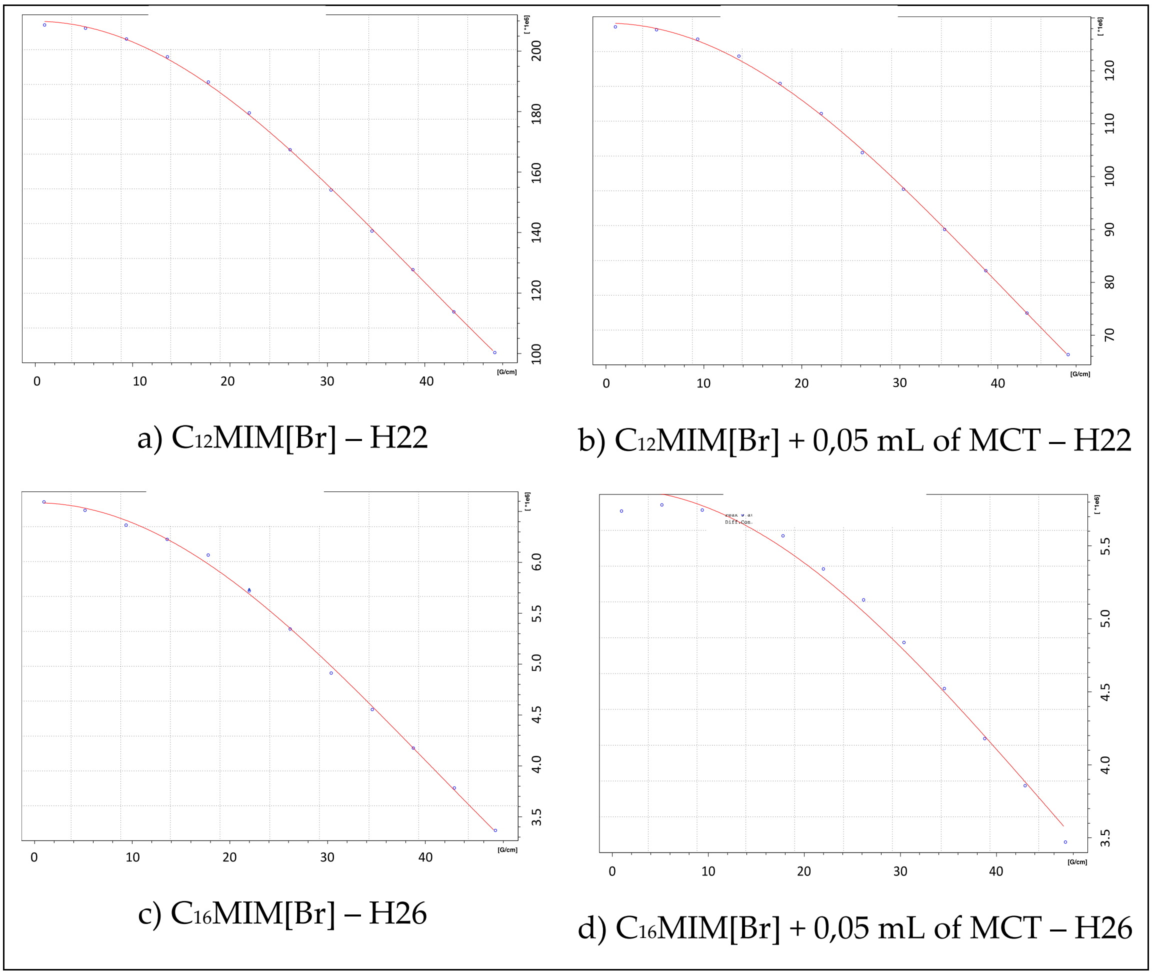

Figure S33. DOSY graph for (a) C_{12}MIM[Br] – H22; (b) C_{12}MIM[Br] + 0,05 mL of MCT – H22; c() C_{16}MIM[Br] – H26; (d) C_{16}MIM[Br] + 0,05 mL of MCT – H26.

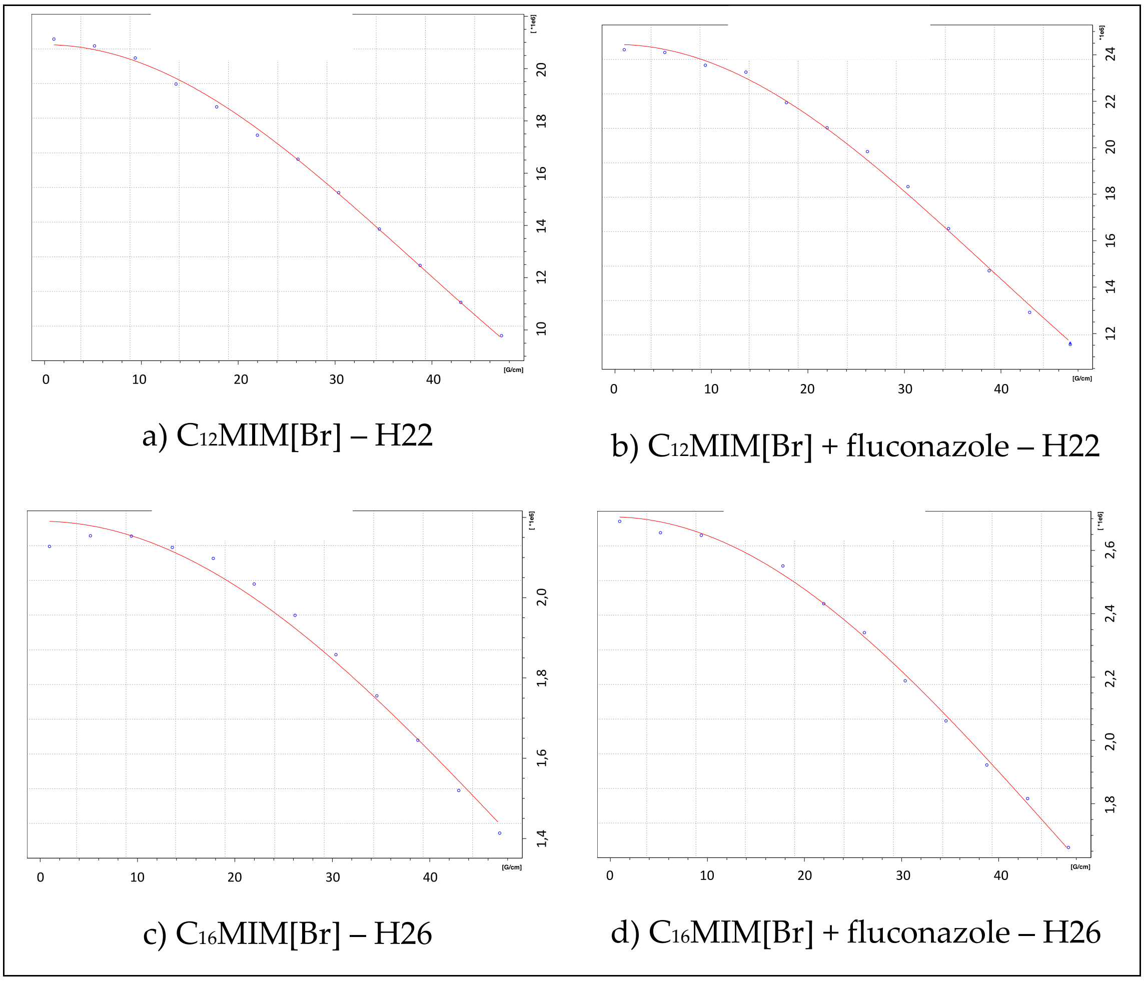

Figure S34. DOSY graph for (a) $C_{12}MIM[Br]$ – H22; (b) $C_{12}MIM[Br]$ + fluconazole – H22; (c) $C_{16}MIM[Br]$ – H26; (d) $C_{16}MIM[Br]$ + fluconazole – H26.

5. Images of emulsions with ILs.

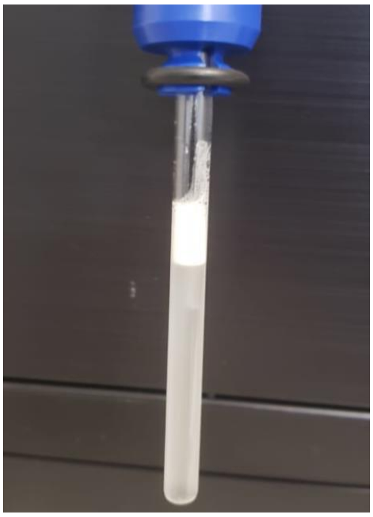

(a)

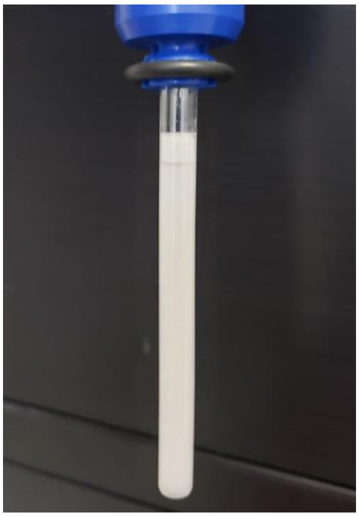

(b)

Figure S35. Images of emulsions (a) $C_{12}MIM[Br]$ + 0,15 mL of MCT; and (b) $C_{16}MIM[Br]$ + 0,15 mL of MCT; in D_2O.
